# Isolipoic acid-linked gold nanoparticles bearing the thomsen friedenreich tumor-associated carbohydrate antigen: Stability and *in vitro* studies

**DOI:** 10.3389/fchem.2022.1002146

**Published:** 2022-10-10

**Authors:** Utpal K. Mondal, Joseph J. Barchi

**Affiliations:** Chemical Biology Laboratory, Center for Cancer Research, National Cancer Institute at Frederick, Frederick, MD, United States

**Keywords:** Thomsen-Friedenreich antigen, tumor-associated carbohydrates, gold nanoparticles, galectin-3, multivalency

## Abstract

We have previously prepared gold nanoparticles (AuNPs) bearing the Thomsen-Friedenreich antigen disaccharide (TF_ag_), a pan-carcinoma, Tumor-Associated Carbohydrate Antigen (TACA), as tools for various assays and biological applications. Conjugation to AuNPs typically involves the use of thiols due to the affinity of sulfur for the gold surface of the nanoparticle. While a use of a single thiol-containing ligand bound to the gold surface is standard practice, several studies have shown that ligands bearing multiple thiols can enhance the strength of the conjugation in a nearly linear fashion. (*R*)-(+)-α-Lipoic acid (LA), a naturally occurring disulfide-containing organic acid that is used as a cofactor in many enzymatic reactions, has been used as a linker to conjugate various molecules to AuNPs through its branched di-thiol system to enhance nanoparticle stability. We sought to use a similar system to increase nanoparticle stability that was devoid of the chiral center in (*R*)-(+)-α-lipoic acid. Isolipoic acid, an isomer of LA, where the exocyclic pentanoic acid chain is shifted by one carbon on the dithiolane ring to produce an achiral acid, was thought to act similarly as LA without the risk of any contaminating (*L*)-(−) isomer. We synthesized AuNPs with ligands of both serine and threonine glycoamino acids bearing the TF_ag_ linked to isolipoic acid and examined their stability under various conditions. In addition, these particles were shown to bind to Galectin-3 and inhibit the interaction of Galectin-3 with a protein displaying copies of the TF_ag_. These agents should prove useful in the design of potential antimetastatic therapeutics that would benefit from achiral linkers that are geometrically linear and achiral.

## 1 Introduction

Tumor-Associated Carbohydrate Antigens (TACAs) are glycan structures covalently attached to proteins or lipids in various forms on the surface of tumor cells (Feizi and Childs, 1985; Hakomori, 1991; Dabelsteen, 1996). They differ from the normal cell glycan repertoire insofar as the tumor biosynthetic machinery is modified *via* a disparate regulation of glycosyltransferases and hydrolases. This produces aberrant and distinct cell-surface glycan structures that are unique to tumors, and these structures impart modified biophysical and protein binding characteristics to individual tumor types. In addition, some of these tumor-associated glycans can be recognized as “non-self” by the immune system (hence the moniker, “antigen”) eliciting both humoral and (sometimes) cell-mediated responses (Andreana, 2009). As a result, there have been myriad attempts to prepare vaccine constructs to raise effective and durable immune responses to TACAs (Toyokuni and Singhal, 1995; Xu et al., 2005; Franco, 2008; Guo and Wang, 2009; Liu and Ye, 2012; Yin and Huang, 2012; Amon et al., 2014; Feng et al., 2016; Wei et al., 2018; Jin et al., 2019). In addition, some of these TACAs are also ligands for carbohydrate-binding proteins (CBPs) that are involved in cell adhesion as they relate to cancer progression and metastasis. Thus, inhibition of these interactions is a way to interrupt tumor aggressiveness and metastatic spread.

One particular TACA that has been used in both of the aforementioned therapeutic arenas is the Thomsen Friedenreich antigen (TF_ag_), which is the core 1 O-linked disaccharide Galβ1-3GalNAc found primarily attached in an α-*O*-linkage to the hydroxyl group of serine and threonine residues of various cell-surface proteins, primarily mucins. TF_ag_ is a classically truncated O-glycan found on many tumors of the breast, prostate and pancreas. The TF_ag_ has been the subject of a wealth of anticancer therapeutic design, either through inhibition of its binding to cancer-relevant proteins (Peletskaya et al., 1997; Khaldoyanidi et al., 2003; Jeschke et al., 2006; Jeschke et al., 2007; Gao et al., 2012; Glinskii et al., 2012; Poiroux et al., 2017; Hoffmann et al., 2020) or as an immunogen in various vaccine design strategies (Slovin et al., 2005; Awad et al., 2012; Brinas et al., 2012; Gaidzik et al., 2013; Ulsemer et al., 2013; Yi et al., 2013; Bourgault et al., 2014; Johannes et al., 2015; Son et al., 2016; Sun et al., 2016; Trabbic et al., 2016; Flechner et al., 2019; Trabbic et al., 2019; Wu et al., 2019; Kleski et al., 2020; Wu et al., 2021; Berois et al., 2022). Mechanistically, the TF_ag_ has been unequivocally shown to interact with the CBP Galectin-3, a β-galactoside binding protein that interacts both intra- and extracellularly with many glycoproteins, is overexpressed in a variety of tumors and whose expression is directly correlated with tumor aggressiveness and metastasis (Takenaka et al., 2002). TF_ag_ is involved in adhesion of tumor cells to the endothelium and these interactions can mediate signaling that allows extravasation of primary tumor tissue (Glinsky et al., 2001; Khaldoyanidi et al., 2003; Glinsky, 2006; Yu et al., 2007; Compagno et al., 2014; Hauselmann and Borsig, 2014; Xin et al., 2015). Thus, inhibitors of the Gal-3/TF_ag_ interaction are potential therapeutics in several types of cancers. TF_ag_ analogues (Glinskii et al., 2012; Santarsia et al., 2018), truncated portions of Gal-3 (John et al., 2003), TF_ag_-mimicking peptides (Glinsky et al., 2000; Newton-Northup et al., 2013) and natural glycopeptides containing multiple copies of TF_ag_ (Guha et al., 2013) have all been shown to inhibit this interaction in different cell-based systems. In addition, antibodies to the TF_ag_ have been used as anticancer agents, especially against breast and prostate tumors (Glinsky et al., 2001; Glinsky et al., 2003; Tantivejkul et al., 2004). JAA-F11, a monoclonal antibody (mAb) to α-TF_ag_ (Rittenhouse-Olson, 2007), has shown good *in vitro* and *in vivo* activity in breast tumor models, with inhibition of spontaneous metastasis (Heimburg et al., 2006), distinct tumor staining of TF_ag_-positive tissue (Ferguson et al., 2014; Karacosta et al., 2018) and it has been humanized for potential clinical development (Tati et al., 2017). In our own lab, we have developed and commercialized an antibody to a TF_ag_-containing peptide sequence from the mucin MUC4, a biomarker for pancreatic cancer, that is highly selective for tumor tissue and binds metastatic foci in tissue arrays (Trabbic et al., 2019).

Monovalent carbohydrate-protein interactions (CPIs) are known to be inherently weak and are strengthened by multivalency (the “Velcro effect”) (Nicotra et al., 2014). Cell-surface CPIs utilize multimeric copies of protein and sugar to fine tune the strength of a specific interaction, and this is also the case for TF_ag_-Gal-3 (or other CBP) binding events. Multivalent TF_ag_ constructs have been pioneered by the research group of Roy and coworkers (Baek et al., 2001; Baek and Roy, 2002; Roy and Baek, 2003). These studies showed that dendrimers and saccharide polymers of the TF_ag_ can potentiate the interaction of the disaccharide with proteins by several orders of magnitude. Our group has made inroads into the use of gold nanoparticles (AuNPs) for the multivalent presentation of TF_ag_ and TF_ag_-containing glycopeptides, and showed that these constructs can bind to Galectin-3 (Gal-3) and inhibit Gal-3 interactions with other proteins. (Svarovsky et al., 2005; Sundgren and Barchi, 2008; Barchi, 2011; Brinas et al., 2012; Glinskii et al., 2014; Biswas et al., 2015). In addition, we have performed several antimetastatic *in vivo* studies in our lab with many of our constructs that early on seemed quite promising, but results have been inconsistent (and hence unpublishable). We reasoned that the stability of the TF_ag_ ligand on the AuNP may be an issue when these AuNP are in the systemic circulation in an intact animal. In an attempt to address this, we set out to make a similar construction, with a highly stable and symmetric linker that could be a common reagent for all subsequent nanoparticle syntheses. Comprehensive stability and *in vitro* studies suggested that this linker strategy is much more desirable than previous designs and will be useful in future therapeutic design strategies.

Development of novel nanomaterials with 3-dimensional self-assembled monolayers of gold (AuNPs) has been an incredibly active area of research in the past two decades. A wide array of these constructions has been designed and synthesized for therapeutic applications against a variety of diseases. In a majority of studies, the synthesis of AuNPs has followed the classic Turkevich (Turkevich et al., 1951; Enustun and Turkevich, 1963) or Brust methods (Brust et al., 1994; Brust et al., 1995). Attachment to the gold surface is usually mediated *via* a thiol functionality due to the high affinity of Au for sulfur atoms. Several studies have shown that additional means of attachment, through molecules containing 2–3 conjugatable sulfur atoms increases the strength of binding as each S-atom can contribute a defined bond strength in an additive way. (Park et al., 2005; Wojczykowski et al., 2006). This effect is a form of “multivalency” and the added stabilization prevents simple place exchange reactions with various concentrations of added soluble thiols.

**FIGURE 1:**
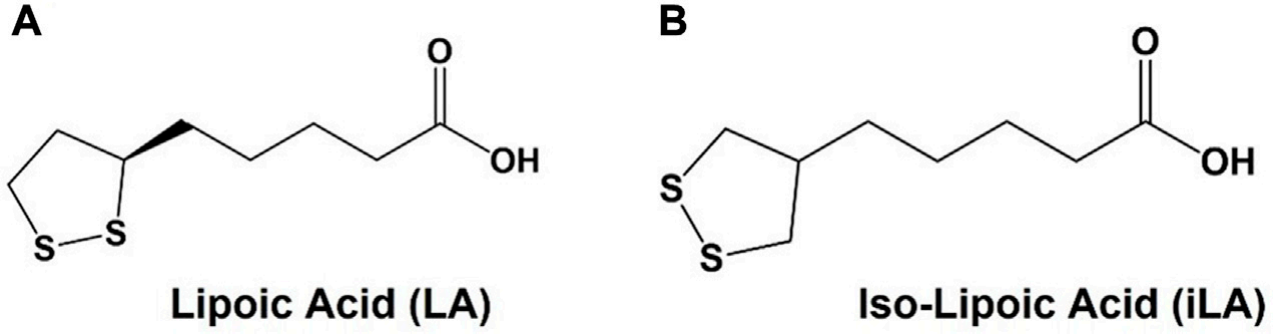
**(A)** Structures of lipoic (LA) and **(B)** iso-lipoic acid (iLA).

One molecule that has garnered substantial attention as a bidentate ligand for AuNP conjugation is α-lipoic acid (LA), sometime referred to as thioctic acid (Figure 1). LA is a naturally occurring cofactor for endogenous enzymatic functions and is sold as a dietary supplement for its antioxidant properties (Goraca et al., 2011). Other than AuNPs, LA has been used in countless applications as a coating for a variety of other nanoparticles, such as nanospheres, cross-linked polymers and quantum dots (Koufaki et al., 2009; Jin et al., 2020), as well as for the preparation of glyco-AuNPs (Yeh et al., 2021) The additional stability, ready availability and ease of use have been the primary features that led to LA’s popularity. In addition to the convenient cyclic disulfide for attachment to metallic nanoparticles, LA has a “built in” conjugatable carboxylate group that is used for simple peptide bond coupling to most any molecular family of ligands, antigens, lipids or nucleic acids. LA has one chiral center and is often used as a racemic mixture. The (R)-(+)-isomer is the naturally occurring enantiomer, but many commercial preparations of “chiral” LA may contain small amounts of the (S)-(−)-isomer. An achiral analogue that retains similar conjugation bond strengths would be desirable in applications where enantiomeric purity is desire. In 2010, Tucker, et al., published an improved synthesis of isolipoic acid (*iso*-LA, Figure 1B) an achiral analogue of LA where the sidechain was shifted to the central carbon of the disulfide-containing 5 membered ring (Joly et al., 2010). This symmetrical molecule suggested a novel way of attaching ligands or other molecular families to nanoparticles through a “dual-pronged” approach. We present the synthesis and evaluation of TF_ag_-coated AuNPs with a new easily prepared linker based on *iso*-LA.

## 2 Results

### 2.1 Synthesis

**SCHEME 1:**
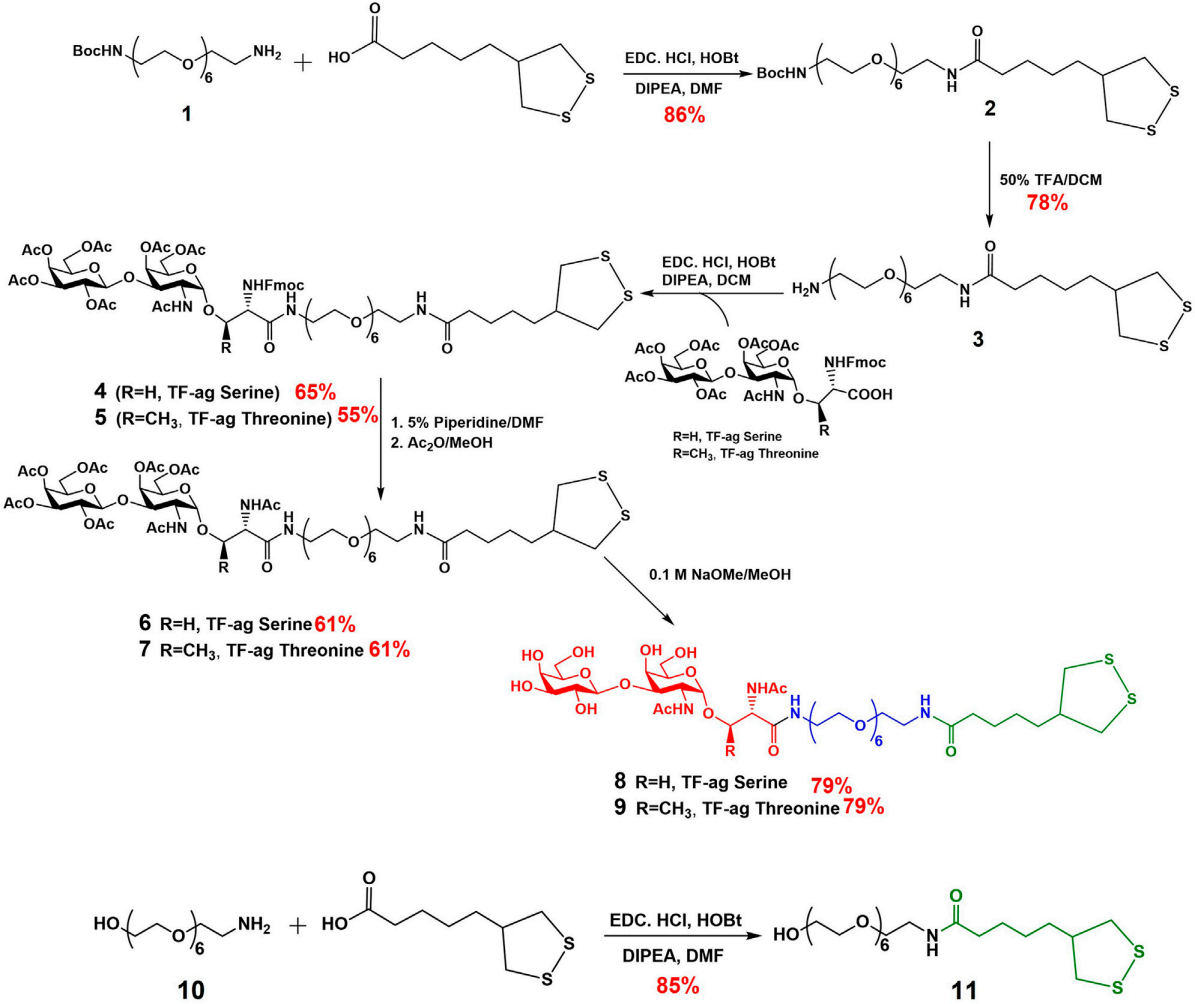
Synthesis of TF-ag Conjugates (**8** and **9**) and Control ligand (**11**).

Previous syntheses of TF_ag_-containing AuNPs in our lab were built around a TF_ag_ glycoamino acid and a specific linker strategy that we developed previously for our AuNP-based vaccine design (Brinas et al., 2012). Modifications to the synthesis were made to accommodate the linkage to a serine or threonine amino acid (Biswas et al., 2015). A very simple adjustment was made to build the linker after the preparation of large amounts the *iso*-LA precursor (Scheme 1). The yields in all steps of the *iso*-LA precursor were consistent with literature values. Following a similar protocol to one we published previously, the *iso*-LA derivatized hexa-PEGylated linker was prepared from commercially available mono-Boc-protected diamine PEG compound **1** and *iso*-LA *via* simple EDC/HOBt-mediated peptide coupling followed by Boc deprotection and repeated peptide coupling to attach the TF_ag_-conjugated Fmoc-glycoamino acids of both serine and threonine. Removal of the Fmoc group, N-acetylation and Zemplen deprotection of the O-acetyl groups yielded compounds **8** (Serine derivative) and **9** (Threonine derivative) as precursors to nanoparticle synthesis. An *iso*-LA-linked hexaPEG conjugate terminated by a hydroxyl group was also prepared as a “control” ligand by simply coupling another commercially available unprotected hydroxyl-terminated PEG amine **10** with *iso*-LA to give compound **11**. All steps were high-yielding and all new compounds were purified by reverse phase HPLC and characterized by high resolution mass spectrometry and multidimensional NMR techniques.

**FIGURE 2:**
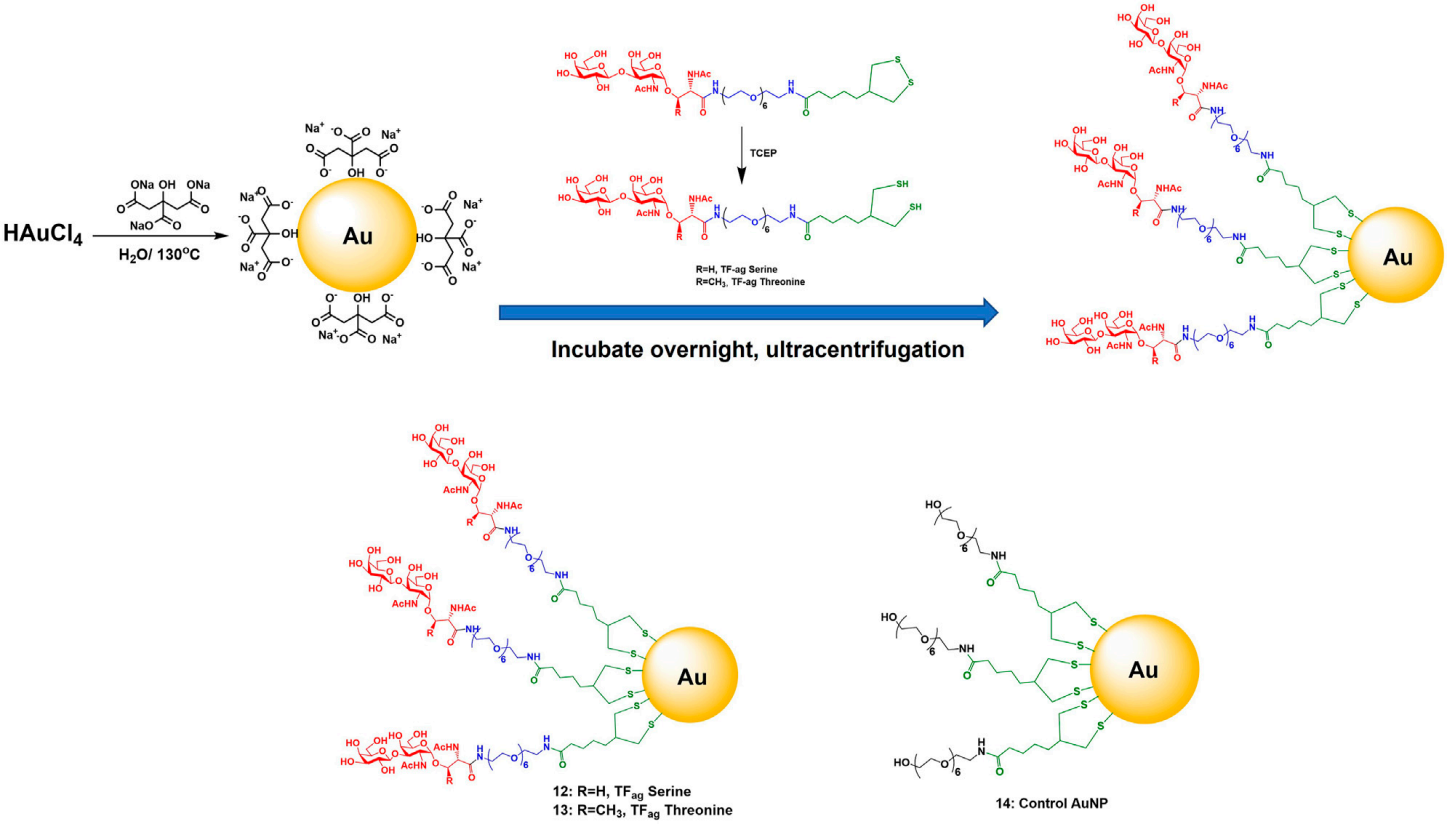
Synthesis of AuNPs prepared in this work and structure of AuNPs **12–14**.

The synthesis of AuNPs proceeded from previously prepared solutions of AuNPs of defined size made *via* the Turkevich method (Figure 2). Concentrations were such to yield particles in the 16 nm core diameter size range. Solutions of these citrate stabilized AuNPs were analyzed for gold content by Inductively Coupled Plasma Mass Spectrometry (ICP-MS) which allowed calculation of accurate nanoparticle concentrations. Simple place exchange reactions allowed coupling of the TF_ag_-containing and control ligands to be attached to the citrate-stabilized (“naked”) AuNPs after brief treatment with immobilized Tris [2-carboxyethyl] phosphine (TCEP) resin to reduce the cyclic disulfide bond. AuNPs **12–14** were purified by concentrating solutions on 50K cutoff filter membranes followed by resuspension and washing with MilliQ water. Nanoparticles were lyophilized and fully characterized as described in Section 2.2.

### 2.2 Characterization

While there are now thousands of reports on the synthesis and biomedical applications of AuNPs, characterization data of individual systems is often highly variable and sometimes incomplete. Thus, we set out to perform comprehensive assessments of all of our AuNPs by a variety of methods to ensure quality control and establish a “Lab Standard” for AuNP characterization.

**FIGURE 3:**
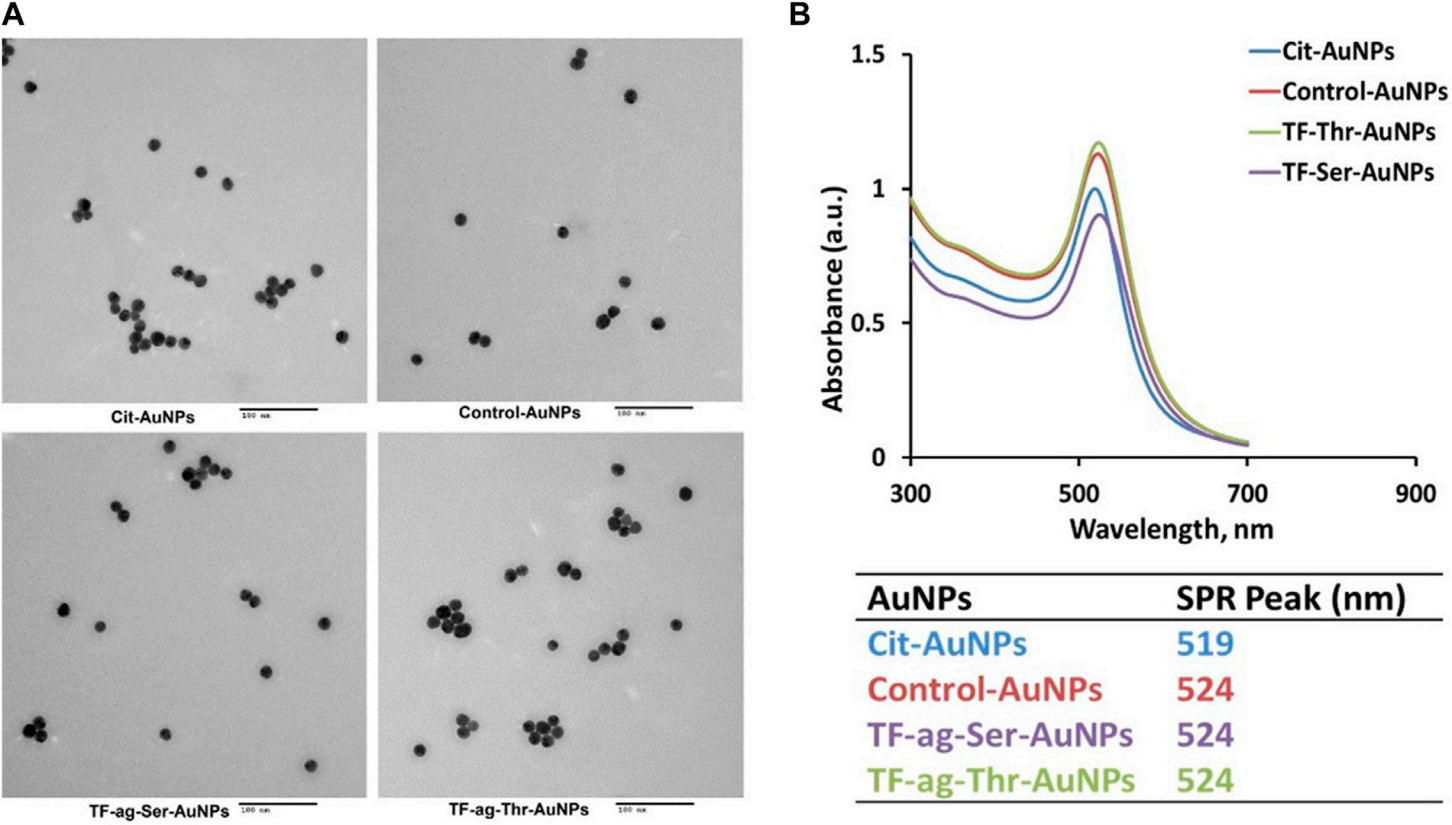
**(A)** Transmission electron micrographs of citrate-stabilized AuNPs and of AuNPs **12–14**. **(B)** Plasmon band UV/Vis spectra of same AuNPs as in A, along with table of SP maximum for each material.

**Transmission Electron Microscopy (TEM)**. All precursor solutions were examined by TEM and showed highly uniform core sizes with narrow size distribution histograms (Figure 3A and Supplementary Figure S1). Liganded nanoparticles met identical criteria for uniformity and size consistency.

**UV-Vis Surface Plasmon Band**. Three-dimensional self-assembled spherical AuNPs are known to interact with light at specific wavelengths to cause oscillation of the free electrons at the gold surface; these oscillations are in resonance with the incoming light frequency, resulting in an absorption/emission spectrum where the absorption is in the 520 nm range. Emission is dependent on size and shape and is often in the red spectrum (700 nm). Citrate stabilized AuNPs of 16 nm have a maximum at 520 nm; addition of ligand causes a slight but consistent red shift to 524 nm (Figure 3B). This is also indicative of particle dispersiveness whereas aggregation cause a loss of signal and is characteristic of agglomeration (*vide infra*).

**MALDI Mass Spectrometry**. There have been several studies that have used MALDI mass spectrometry to study the morphology and the ligand distribution of AuNPs (Luo et al., 2017). MALDI was used to determine the presence of ligand on the particles. Supplementary Figure S2 shows the MALDI mass spec data for each of the AuNPs X-Y. Each spectrum contains peaks from the sodium adducts of each ligand that was coated on the gold core.

**Dynamic Light Scattering (DLS)**. Hydrodynamic diameters were determined by DLS. All materials prepared gave quite uniform and single peak in the spectrum (Supplementary Figure S3). Intensity and volume measurements were similar where very few larger particles were observed in the intensity data. All correlation functions converged and thus the uniformity of the AuNPs were very high with Polydispersity Indices (PDI) all below 0.1 (Table 1).

**TABLE 1:** Physical properties of materials prepared in this work.

AuNPs	Avg Hydrodynamic Diameter by DLS (nm)	Core Size by TEM (nm)	PDI	Zeta Potential (mV)
Cit-AuNPs	23.9 ± 4.28	16.14 ± 1.88	0.034	−35.4 ±7.63
Control- AuNPs	25.3 ± 7.60	16.27 ± 1.62	0.097	−15.1 ±4.64
TF-ag-Ser- AuNPs	24.6 ± 4.90	16.34 ± 1.71	0.061	−17.3 ±3.50
TF-ag-Thr- AuNPs	24.7 ± 6.46	16.36 ± 1.48	0.049	−16.7 ±3.20

**Zeta Potential**. Citrate-stabilized AuNPs are coated with anionic citrate ions that impart a highly negative zeta potential that is partially neutralized by the addition of neutral organic ligand layers, such as the ones used in this study. This was the case here as the zeta potential adjusts from −35 mV for the naked particles to between (−15)–(−17) mV for the coated particles. This drop in voltage did not have any detrimental effect on the stability or aggregative properties of the prepared AuNPs (*vide infra*).

**Quantitation of TF_ag_ Disaccharide on AuNPs**. The copy number of the serine and threonine-linked TF_ag_-containing ligands on the AuNPs were quantitated by the well-known Phenol-Sulfuric acid method using a standard curve with varying concentration of β-lactose. Table 2 shows this number to be close to 1,600 for each nanoparticle prepared. This number was consistent among different batches and the copy number was essentially identical for each ligand, suggesting that the choice of amino acid conjugate does not alter the place exchange process. Table 2 also shows the average occupied surface area for each ligand as calculated from the core diameter and known surface properties of the gold 3-dimensional self-assembled monolayers.

**TABLE 2:** Copy number and surface occupancy of ligands on the TF_ag_-containing AuNPs.

AuNPs	Conc. of TF-ag conjugate per 100 μ of AuNP (μM)	Number of TF-ag conjugate per AuNP	Average Occupied Surface Area on AuNP (nm^2^)
TF-Ser-AuNPs	61 ± 0.0015	1620 ± 41	0.51
TF-THr-AuNPs	63 ± 0.0012	1660 ± 32	0.50

**FIGURE 4:**
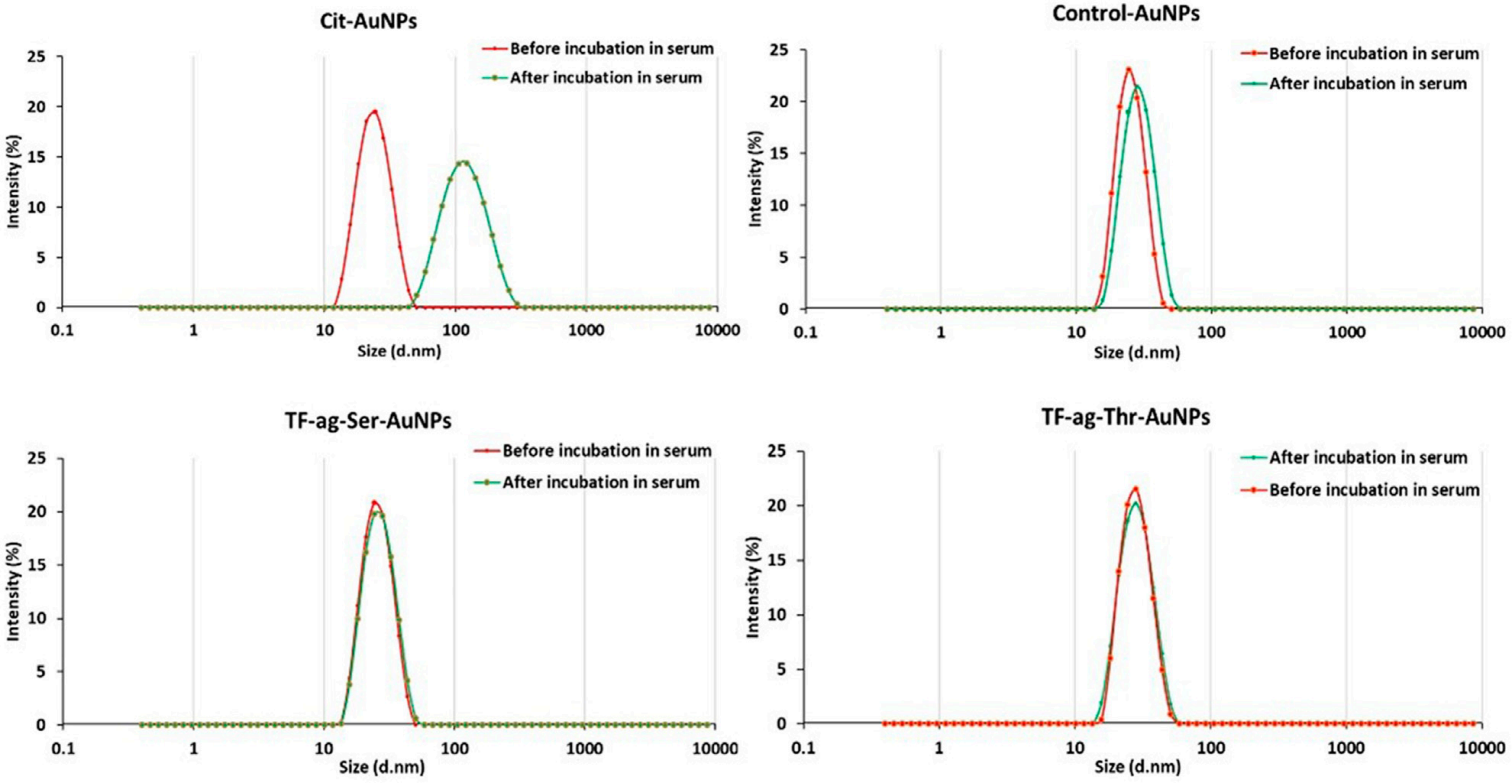
Stability in human serum as analyzed by Dynamic Light Scattering.

**AuNP Stability in high salt and human serum**. The UV plasmon band of each prepared AuNP was monitored in sodium chloride solutions from concentrations ranging from 0 to 1-M salt. The known aggregation of citrate-stabilized nanoparticles, indicated by the loss of this band at 520 nm, occurs at around 50 mM NaCl, whereas of AuNPs **12–14** show no loss of UV absorption in this region over the entire salt concentration range (Supplementary Figure S4). Similar behavior was observed in human serum. Equal volumes of AuNPs and human serum were incubated at 37°C. After 24 h, the AuNPs were pelleted by centrifugation, the supernatant discarded and the AuNPs were re-dissolved in MilliQ water and the hydrodynamic diameter measured by DLS. Figure 4 shows very little, if any, change in size distribution after incubation with serum, suggesting that the nanoparticles will maintain their physical properties and not aggregate if used in an intact organism.

### 2.3 *In vitro* biological activity

Binding to Galectin-3. As mentioned previously, the binding of Gal-3 to the TF_ag_ is an important functional interaction in the metastatic spread of various tumor cell types. Both homotypic adhesion of tumor cells to form clusters and the subsequent binding of these to the endothelium prior to extravasation and tumor spread have both been shown to be mediated by the TF_ag_-Gal-3 interaction (Glinsky et al., 2001; Takenaka et al., 2002; Glinsky et al., 2003; Yu et al., 2007; Zhao et al., 2010; Glinskii et al., 2012; Reticker-Flynn and Bhatia, 2015; Rodriguez et al., 2015). We evaluated our particles in three *in vitro* assays in binding to Gal-3: 1) aggregation assay, 2) direct binding assay and 3) competitive inhibition assay.

First, Aggregation of Gal-3 by the particles was performed by addition of varying concentrations of Gal-3 to a defined concentration of AuNPs and recording both absorption spectra and DLS size measurements vs. time (Supplementary Figures S5, S6). Aggregation is indicated by an obvious increase in hydrodynamic diameter of the particle solution and a dramatic red shift of the plasmon band in the UV spectrum (Boden et al., 2017) (Otten et al., 2016). Both the serine and threonine-coated AuNPs caused rapid and complete aggregation of Gal-3 where the control particles showed no activity at any concentration, indicating that a multivalent display of the TF_ag_ structure is critical and that simply a random display of hydroxyl groups is incapable of causing agglomeration. Plots of absorbance at 700 nm as well as size increase vs. time from DLS data were used to calculate the kinetics of aggregation (Hill function in GraphPad/Prism). The apparent calculated K_d_ values of aggregation were 116 nM and 101 nM for AuNPs **12** and **13**, respectively.

**FIGURE 5:**
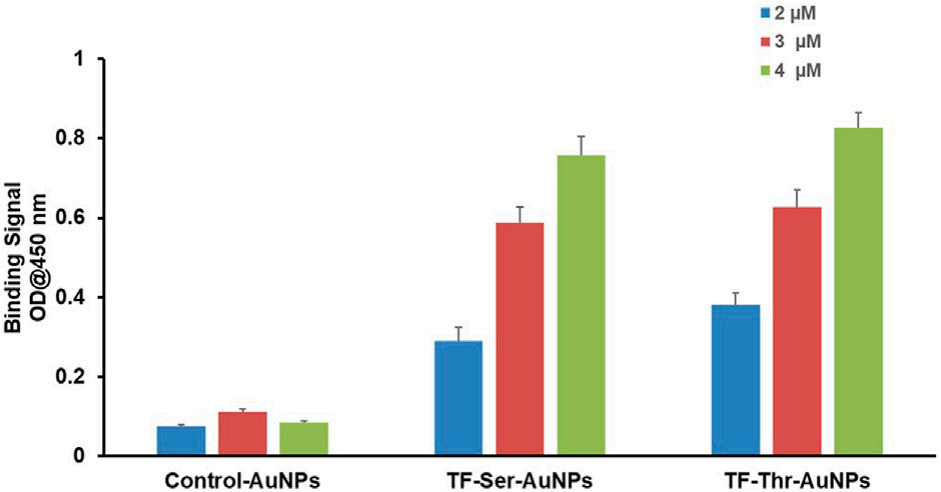
Binding of Gal-3 to AuNPs by simple ELISA experiments with AuNP-coated 96-well plates.

Second, direct binding was measured by simple ELISA where the AuNPs themselves were coated on 96 well plates. A small range of concentrations of Gal-3 were examined and the data is shown in Figure 5. A clear dose response was observed even though the concentrations studied only covered a narrow range (2 μM–4 μM). Both AuNP’s **12** and **13** displayed almost identical binding, while the control AuNPs **14** were completely inactive. The relatively large increase in optical density over this short range of Gal-3 concentrations suggest a multivalent mechanism is operational in this binding event.

**FIGURE 6:**
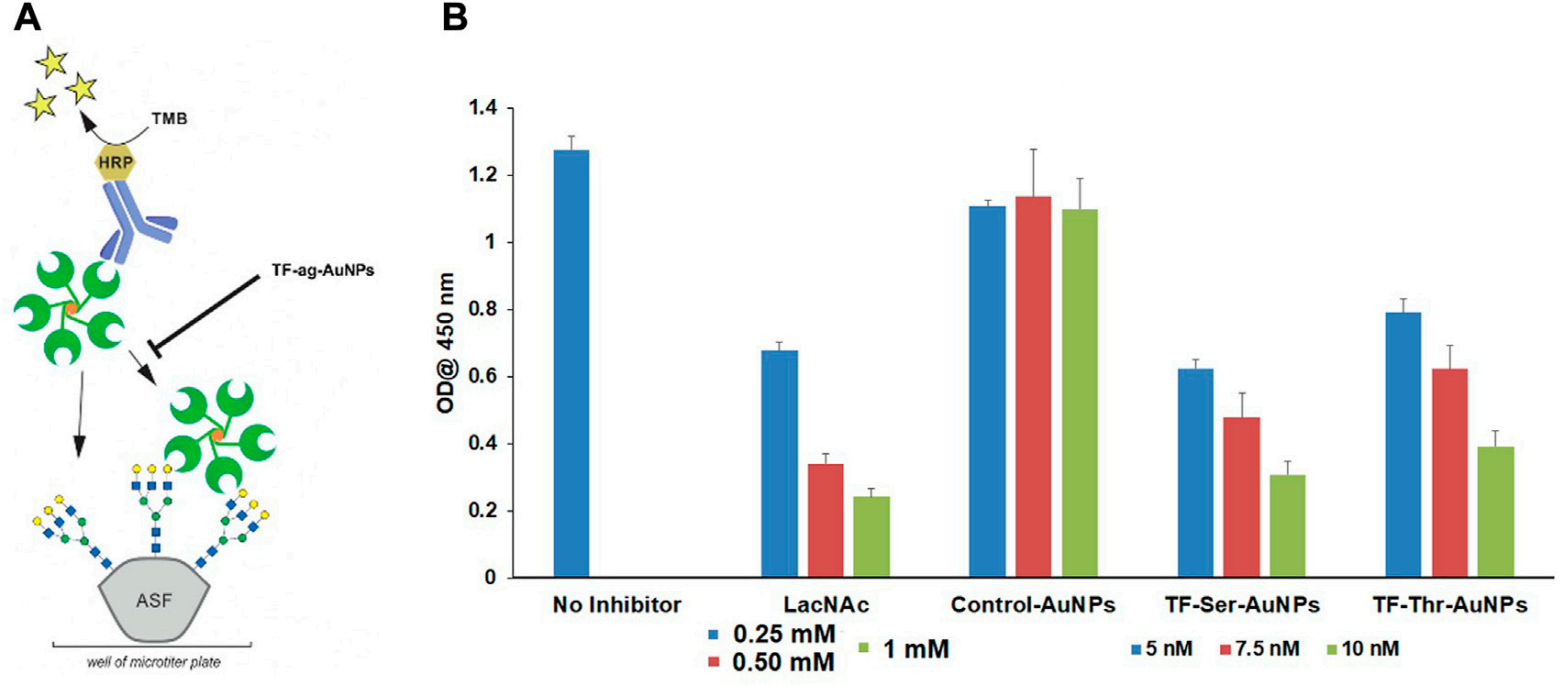
Inhibition of Gal-3 binding to ASF by TF_ag_-coated AuNP. **(A)** Schematic depiction of the assay as shown in reference (Otten et al., 2016). **(B)** Bar graph depicting inhibition of Gal-3 binding to ASF by compounds materials **12–14**.

Third, we performed a competitive binding assay to assess whether the AuNPs would inhibit the binding of a known ligand for Gal-3 (Bumba et al., 2018). Thus, we examined the behavior of nanomaterials **12–14** as inhibitors of the binding of Gal-3 to asialofetuin (ASF). ASF contains several complex N-linked glycans whose branches are terminated by N-acetyl-lactosamine (LacNAc) units, and hence has been employed previously as a multivalent “carrier” of LacNAc. (Dam et al., 2005). Endogenous LacNAc is considered a natural ligand for many galectin family proteins. Inhibitory studies were carried out by treating ASF-coated 96 well plates with Galectin-3, with or without compounds **12–14**; LacNAc was used as a positive control. Figure 6 shows that AuNPs **12** and **13** inhibit the binding of Gal-3 to ASF at low nanomolar concentrations, whereas control AuNPs **14** were again inactive. Inhibition by LacNAc showed a similar trend as our nanoparticles, although at concentrations that were more than 3 orders of magnitude higher, again arguing for the multivalent binding/inhibitory effect of the AuNPs.

### 2.4 Discussion

AuNPs have been prepared in many different guises and used in a variety of biomedical applications for more than two decades. Even with their relatively low toxicity, ability to be made biocompatible and the chemistry available to coat them with any family of molecule, there is yet to be any true AuNPs approved for clinical use in therapeutic arenas such as cancer. Continued research into the development of AuNPs that possess the necessary properties for translation should foster FDA approval of selected metal-based nanomaterials.

In this work we developed a new linker strategy for preparation of AuNP tools for functional studies that hopefully could lead to their use as anticancer therapeutics. The use of lipoic acid has been a mainstay for “dual-pronged” bis-thiol attachment to gold and other metallic surfaces, while “*iso*lipoic” has only been used in one other instance for attachment to a gold surface (Joly et al., 2010). Our reasoning to use of *iso*-LA in place of LA for the preparation of functional AuNPs was twofold: 1) The achiral nature of *iso*-LA solves the issue of “contamination” by the unnatural isomer of LA, thus preventing any unwanted immune responses for use in any *in vivo* setting and 2) Positioning the sidechain in the center of the dithiolane ring produces a linker with a linear directionality from the nanoparticle core, which may relieve any unwanted crowding of ligands like those with LA linkers that protrude from the surface at a distinct angle.

Similar to LA linkers, our TF_ag_ ligands were attached more strongly to the AuNP as those with a single thiol end group. Stability studies showed that these particles maintain excellent uniformity under relatively harsh conditions (1.5 M salt, 50% human serum). All physical properties were aligned with well-constructed AuNPs, where solubility and functionality were maintained in all synthetic materials. AuNPs **12–14** were stable for many months at 4°C with minimal signs of flocculation or aggregation. Preliminary bioassay evaluation of these constructs showed binding Gal-3, a lectin that is involved in tumor aggression and metastasis. Inhibition of Gal-3 binding to ASF, a known protein ligand of Gal-3, was also shown to be a function of these AuNPs; the concentrations for this inhibitory effect were much lower than the same level of inhibition by the monomeric disaccharide LacNAc, a known cognate ligand of many galectins. These results suggest that a multivalent effect is operational with these constructs in inhibition of protein-carbohydrate binding. As stated in the introduction, many platforms that display multiple copies of TF_ag_ have been prepared and also function through multivalency. We feel that these particles are unique in that 1) Their stability rivals other particles, even those with covalent conjugation of the ligand, 2) Synthesis is relatively simple and high yielding and 3) A host of other ligands from diverse structural families can be attached in to these platforms in a straightforward manner.

These properties prompted us to perform a large *in vivo* study of AuNPs **12–14** to evaluate their antimetastatic properties in a 4T1 triple negative breast cancer model. Four groups of 14 animals each were treated either with AuNPs **12**, **13** or **14** where the fourth group was treated with PBS. While little toxicity was evident in the animals after AuNP treatment, all groups were very similar with respect to survival, lung metastasis and overall health. While the *in vitro* data suggested some therapeutic effects would translate to antitumor/antimetastatic effects *in vivo*, this disappointing result suggested that either the AuNPs were simply inactive in this model or the model itself is not suitable for this mode of treatment. We are comprehensively evaluating the biodistribution and *in vivo* stability of these materials in hopes to achieve therapeutic efficacy in future *in vivo* studies.

The results herein show that a simple modification of a popular linker can produce AuNPs of excellent stability while maintaining function. Our data show that these reagents are useful for analyzing TF_ag_ binding and inhibition, and, when compared to other AuNPs with LA-based or other bifunctional linkers, can offer clues as to the optimum disposition of ligands for usefulness as analytical or therapeutic tools. Comparisons of various *in vitro* and *in vivo* bioassays between the present *iso*-LA-based AuNPs with those based on other linkers is currently in progress.

## Data Availability

The original contributions presented in the study are included in the article/Supplementary Material; further inquiries can be directed to the corresponding author.
