# Supplementary material for: Isolipoic acid-linked gold nanoparticles bearing the thomsen friedenreich tumor-associated carbohydrate antigen: Stability and in vitro studies
Source: Front Chem. 2022 Oct 10;10:1002146. doi: 10.3389/fchem.2022.1002146 (PMC9588967; doi:10.3389/fchem.2022.1002146)
Supplement: Supplementary file 1 [file DataSheet1.pdf]

Supporting Information

Isolipoic Acid-Linked Gold Nanoparticles Bearing the Thomsen Friedenreich Tumor-Associated Carbohydrate Antigen: Stability and in vitro Studies

Utpal K. Mondal and Joseph J. Barchi Jr.

Chemical Biology Laboratory, Center for Cancer Research, National Cancer Institute at Frederick, Frederick, MD 21702

General Experimental Procedures……………………………………….Pages S2-S5

Figure S1……………………………………………………………………Page S5

Figures S2 – S7……………………………………………………………Pages S6 -S9

Synthesis of iso-Lipoic Acid………………………………………………Pages S9 -S11

Synthesis of TF-Conjugates and Nanoparticles……………………….Pages S12 -S15

Selected NMR Spectra……………………………………………………Pages S16 – 29

References…………………………………………………………………..Page 30

General Procedures and Reagents: Most chemical reagents were purchased from Sigma-Aldrich. Tetrachloroauric Acid was purchased was purchase from Wuhan Golden Wing Indus-try & Trade Co, Wuhan China. Peracetylated TF-Serine glycoamino acid was either prepared as previously described or purchased from Sussex Research, Ottawa, Ontario, Canada. Solvents were dried in a Grubb still percolation system under a nitrogen atmosphere. t-Boc-N-amido-PEG6-amine and Amino-PEG7-alcohol were purchased from BroadPharm, (San Diego, Ca.). Low resolution LC-MS data were collected on a Shimadzu 2020 single quadrapole using a DUIS ionization source operating in alternating positive and negative mode. Chromatography was performed on a Phenomenex Kinetex C18 2.6 uM column using a gradient of 10% – 95% Water/Acetonitrile with 0.1% formic acid with an inline Simadzu SPD-M20A diode array UV/Vis detector. High Resolution Mass Spectrometry was performed on by ESI-LC/MS on an LTQ-Orbitrap-XL LC/MS^n system at 30,000 resolution. A narrow-bore C_{18} column system eluted with CH_3OH/H_2O gradients tailored to the compounds of interest was used for separations. AuNPs were analyzed for their sizes and zeta potential in 10 mM NaCl at 25°C with a Malvern ZetaSizer NanoZS (Malvern Instruments Ltd, Westborough, MA) equipped with a 633 nm laser and a back-scattering detector in an automated mode. Hydrodynamic size of the samples was measured by using the Stokes-Einstein equation. The zeta potential of the samples was calculated from the electrophoretic mobility using the Smoluchowski approximation. Proton (^1H)and carbon (^{13}C) NMR data were collected on either a Bruker NanoBay 400 Mhz spectrometer with a Bruker 2-channel SMART probe or on a Bruker AVANCE III 500 MHz spectrometer with a TCI (^1H, ^{13}C, ^{15}N) cryoprobe at 25°C. Most data were run in 90%/10% H_2O/D_2O. Water suppression was performed using excitation sculpting (1D pulse sequence zgesgp). Analytical purity of all compounds was determined by a combination of High Resolution NMR and Mass spectrometry data, along with High Performance Liquid Chromatography. From the combination of these techniques, purity was determined to be >95%.

Synthesis of citrate-capped gold nanoparticles

Spherical gold nanoparticles were synthesized by using the classical Turkevich method. In a 250 ml round bottom flask, Tetrachloroauric(III) acid trihydrate ($HAuCl_4.3H_2O$), (0.25 mM, 50 mL) was heated to boiling for 10 mins, wherein sodium citrate dihydrate (38.8 mM, 760 µL) was quickly added. The solution turned purple after one minute and eventually turned to wine red after 5 min and the solution was allowed to boil for another 15 mins. The reaction vessel was cooled to room temperature where excess citrate reagent was removed by filtration through 30KDa cutoff Amicon Centrocon units at 6000 rpm for 15 mins (3x). Finally, the nanoparticles were resuspended in 0.1X PBS, pH 7.0 and characterized for size and zeta potential by DLS and SPR band by UV-Vis spectroscopy. Elemental gold (Au) content was determined by Inductively Coupled Plasma Mass Spectrometry (ICP-MS) at the Nanotechnology Characterization Laboratory of the NCI (https://www.cancer.gov/nano/research/ncl). Solution concentrations were determined by UV-Vis of the band at ~520 nm (Extinction coefficient: 9.1X 10^8 $M^{-1}cm^{-1}$).

Functionalization of gold nanoparticles with control and TF-ligands

Control ligand, TF-Ser and TF-Thr conjugates were dissolved in Mili-Q water to prepare respective stock solutions having 1 mM concentration. To reduce the di-sulfide bond, the ligand solutions (600 µL) were then treated with an equivalent amount of Tris[2-carboxyethyl] phosphine (TCEP) resin gel (ThermoFisher) and incubated for 1 hr. Subsequently, the gel was removed by centrifugation and the ligand solutions were used immediately. To a stirring suspension of nanoparticles (OD 2, 2 mL) in a dried microwave vial was added activated ligand stock solution (600 µL) very slowly over 5 min. The reaction mixture was then allowed to stir for

24 h in the dark. Excess ligand was removed by centrifugation using Amicon spin filter at 5000 rpm for 15 mins and nanoparticles were resuspended in MiIli-Q water. The purification cycle was repeated 3x. The nanoparticles were lyophilized, and concentration was determined by using a UV-Vis method.

Characterization of citrate-capped and functionalized gold nanoparticles

All the nanoparticles were characterized using a combination of several techniques. Hydrodynamic size and zeta potentials were determined by a Zetasizer Nano ZS (Malvern Panalytical, UK) using disposable UV-Vis cuvette and disposable Malvern DTS 1060 zeta potential measurement cell respectively. Surface plasmon resonance (SPR) was recorded on a NanoPhotometer® NP80 (Implen GmbH, München, Germany) in the wavelength range of 300-700 nm at room temperature. Elemental gold (Au) content in citrate capped AuNPs was determined by Inductively Coupled Plasma Mass Spectrometry (ICP-MS) at the NCL which was further confirmed by UV-Vis method. The core size, shape and morphology of the nanoparticles were determined via transmission electron microscopy (TEM) on a JEOL JEM1400 electron microscope after drying on a copper grid.

Confirmation of ligand functionalization on AuNPs

Confirmation of ligand loading on gold nanoparticles were confirmed by SPR, NMR and MALDI-TOF techniques. Surface plasmon of functionalized nanoparticles showed bathochromic shifts from 519 nm to 524 nm, indicative of effective functionalization. All the functionalized nanoparticles were resuspended in water and deuterium oxide (9:1) and their water suppression NMR spectra were obtained. Ligand signal of NMR spectra confirmed the surface functionalization of the AuNPs. For MALDI-TOF spectra, α-Cyano-4-hydroxycinnamic acid was used as a matrix. Both the ligands and matrix were dissolved in water and ethanol mixture (1:1) and spotted on a MALDI plate sequentially and allowed to dry for 10 mins. Subsequently, their MALDI-TOF spectra were recorded in the operating voltage of 80 eV using the reflectron mode.

Quantification of TF-ligand loading on gold nanoparticles

Quantification of TF-Ser/Thr ligands loading were determined by a well-known phenol-sulfuric acid method by comparing with a standard curve generated using β-lactose solutions having concentrations in the range of 0.025-0.5 mg/mL. TF-Ser/Thr-AuNPs solutions (50 µL) were first treated with 5 µL of concentrated HCl and incubated in the dark overnight to cleave the sugars from the nanoparticle surface. The resulting hydrolysate/nanoparticles mixture were centrifuged at 14000 rpm for 10 mins and supernatants were taken transferred to glass vials which were treated with 150 µL of concentrated sulfuric acid. After 30 mins incubation period, 30 µL of 5% phenol was added to each vial and heated at 90ºC for 1 h. Once the color developed the reaction solutions were transferred to microwell plates and absorbance was measured at 490 nm using a microplate reader.

Stability of gold nanoparticles in High Salt Solutions

The stability of both the citrate-capped and functionalized nanoparticles in different concentration of NaCl was assessed following a literature procedure with modifications.[1] In a 96-well plate, 50 µL of AuNPs (100 µg/mL) were placed in each well. NaCl dissolved in Milli-Q (2 M) water was added to each well to obtain final concentrations of 0, 12.5, 25, 37.5, 50, 100, 125, 250, 500 and 1000 mM sodium chloride in each well. After 24 h incubation in the dark at room temperature, the UV-Vis spectra (surface plasmon) were recorded from a microplate reader in the wavelength range of 400-700 nm. Changes in color (SPR peak) of AuNPs indicated their aggregation behaviors and thus stabilities.

Stability of gold nanoparticles in human serum

We studied all the nanoparticles for their serum stability by following a.[2] Briefly, both citrate-capped and functionalized nanoparticles (500 µL) were treated with 500 µL of human serum in centrifuge tubes and incubated at 37ºC. After 24 h incubation time, the AuNPs were centrifuged at 14,000 rpm for 10 mins, supernatants were discarded, and the nanoparticles were resuspended in Milli-Q water. The hydrodynamic size was determined by DLS in a Zetasizer Nano ZS.

Binding studies of AuNPs to Gal-3

Binding of functionalized AuNPs to Galectin-3 was reconfirmed by an ELISA assay. The nanoparticles (0.5 mg/mL, 50 µL) were immobilized in F16 Maxisorp NUNC-Immuno Modules (Thermo Scientific, Roskilde, Denmark) for 12 h at 4ºC. After washing three times with PBST (PBS with 0.05% Tween 20), the wells were treated with BSA (3% w/v) to block the residual binding site. The wells were washed again three times with PBST and subsequently different concentration of Galectin-3 (2 µM to 4 µM, 50 µL) were added. After 1.5 h incubation at 37ºC, un-bound galectin-3 was removed by washing three times with PBST and wells were treated with HRP conjugated mouse anti-galectin-3 antibody (1:2000 in PBS, 50 µL) for 1 h at 37ºC. The wells were again washed with PBST three times and TMB One substrate solution was added to initiate the reaction with peroxidase. After 30 min, the reaction was stopped by adding 1 M hydrochloric acid (50 µL). The resulting signal was recorded at 450 nm by using a Biotek plate reader.

Inhibition studies of Gal-3 by AuNPs

Competitive inhibition of galectin-3 by AuNPs were assessed by an ELISA assay following a literature procedure with modifications.[3] N-Acetyl-D-Lactosamine was utilized as a control Galectin-3 inhibitor. Asialofetuin (0.1 µM in PBS, 50 µL), a well-known Galectin-3 binder, was first immoblized in F16 Maxisorp NUNC-Immuno Modules (Thermo Scientific, Roskilde, Denmark) for 12 h at 4ºC. The microwells were then washed with PBST (PBS with 0.05% Tween 20) three times (3X 250 µL). Bovine serum albumin (3% w/v in PBS, 300 µL) was added to each well and incubated for 1 h at room temperature to block residual binding site. After washing with PBST (3X 300 µL), 25 µL of galectin-3 (1 µM in PBS) and 25 µL of AuNPs (5-10 nM) were added to each well simultaneously. After 1 h incubation at 37ºC, un-bound Galectin-3 was removed by washing with PBST three times. Bound Galectin-3 was detected by utilizing HRP conjugated mouse anti-galectin-3 antibody and subsequent reaction with TMB substrate following above mentioned procedure. Binding signal was recorded at 450 nm by using a Biotek plate reader.

Aggregation Studies of AuNPs to Gal-3 by UV-Vis spectroscopy and DLS

Galectin-3 induced aggregation behavior of functionalized AuNPs were studied by following a literature procedure with modifications.[4] Functionalized AuNPs (10 nM in water, 100 µL) were added to each well in a 96-well plate. 100 µL of galectin-3 with varying concentration from 0-10000 nM were added to each well. After 30 min incubation at room temperature the absorption spectrum were recorded (wavelength range 400 to 750 nm,10 nm step) by a plate reader. The absorbance at 700 nm were plotted against concentration of galectin-3 and Kd value of aggregation was determined by fitting the curve to a Hill functions using GraphPad Prism software. Similar experiments were performed with hydrodynamic size measurements over time

as determined by DLS. and hydrodynamic sizes were determined by DLS (after 30 min incubation period with Gal-3).

Aggregation kinetics of AuNPs to Gal-3

Aggregation kinetics of AuNPs to galectin-3 were studied by DLS and UV-Vis Spectroscopy by following literature procedures.[1] To 96 well microplates were added 100 µL of AuNPs (10 nM) followed by Gal-3 in a 1:1 ratio to obtain K_D concentration of galectin-3 (116 nM for TF-Ser-AuNPs and 101 nM for TF-Thr-AuNPs) and 5 nM concentration of AuNPs. For Control-AuNPs, 150 nM concentration of galectin-3 was added to microwell plate. The absorbance at 700 nm was recorded at every minute for 30 min. Similarly, hydrodynamic size was determined at every minute with an acquisition time of 55s for 30 min using ultra-low volume cuvettes.

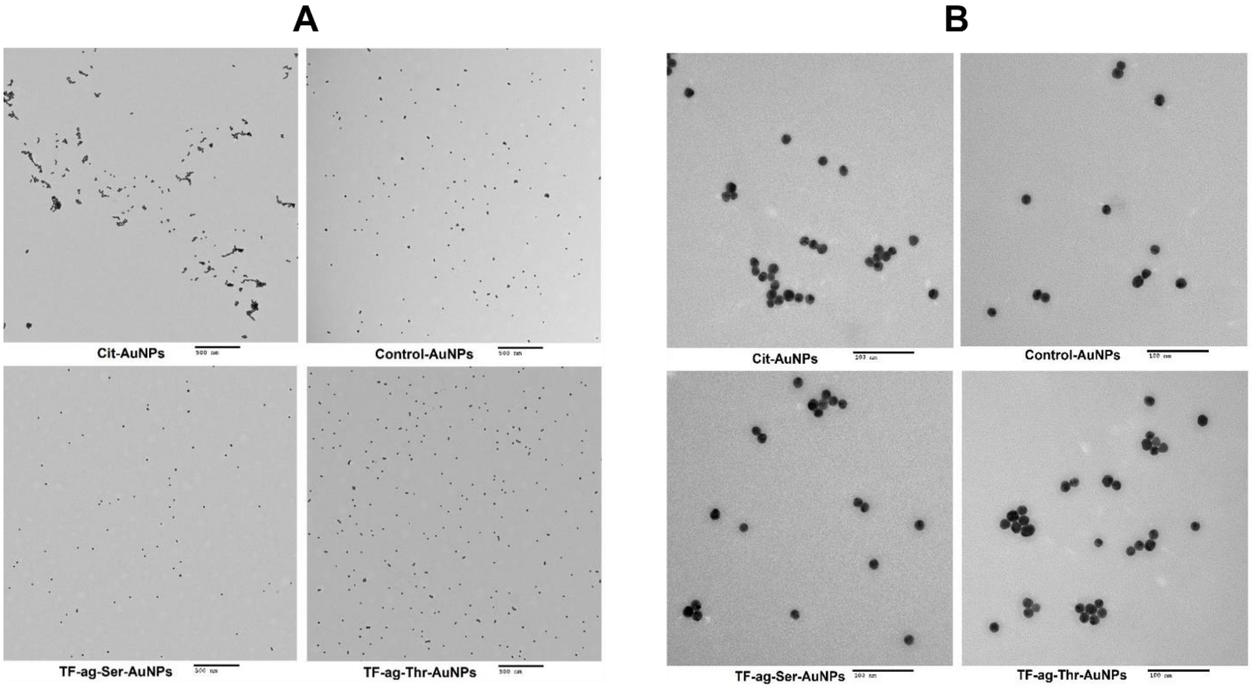

Figure S1. (A) Zoomed out view of TEM images of the initially synthesized AuNPs. (B) Close up regions of all AuNPs showing size and shape uniformity.

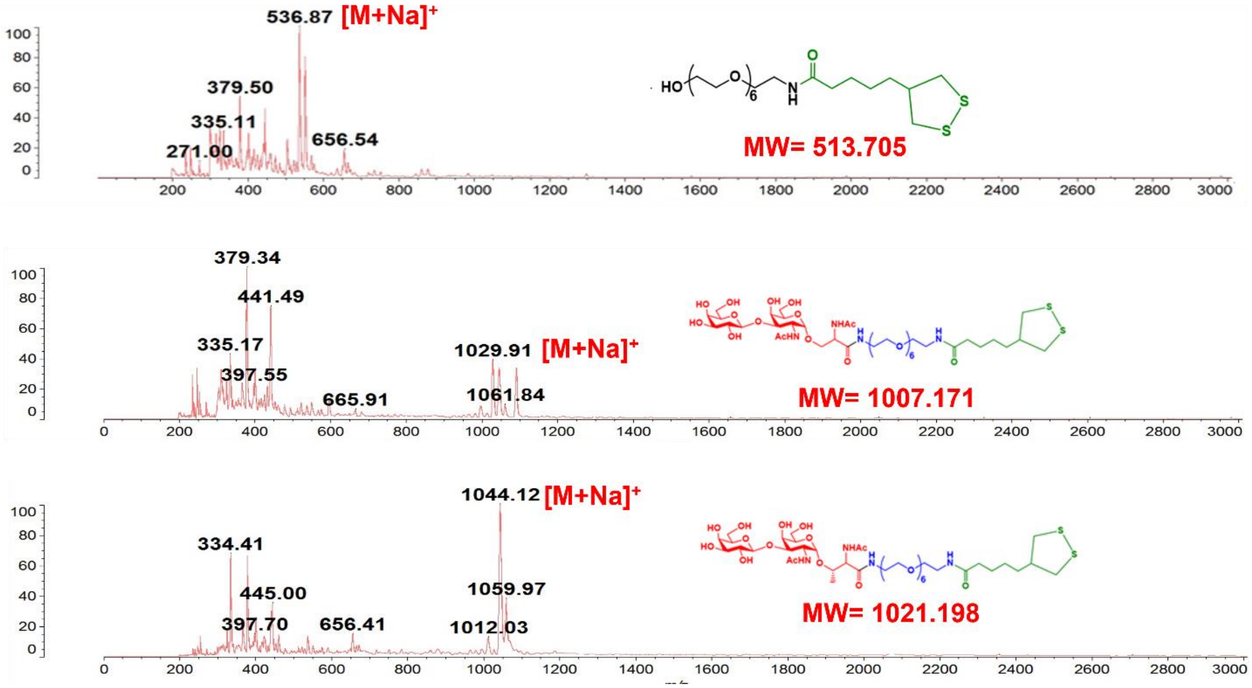

Figure S2. MALDI Mass spec data for freshly synthesized AuNPs with control ligand (top), TF-Ser (middle) and TF-Thr (bottom).

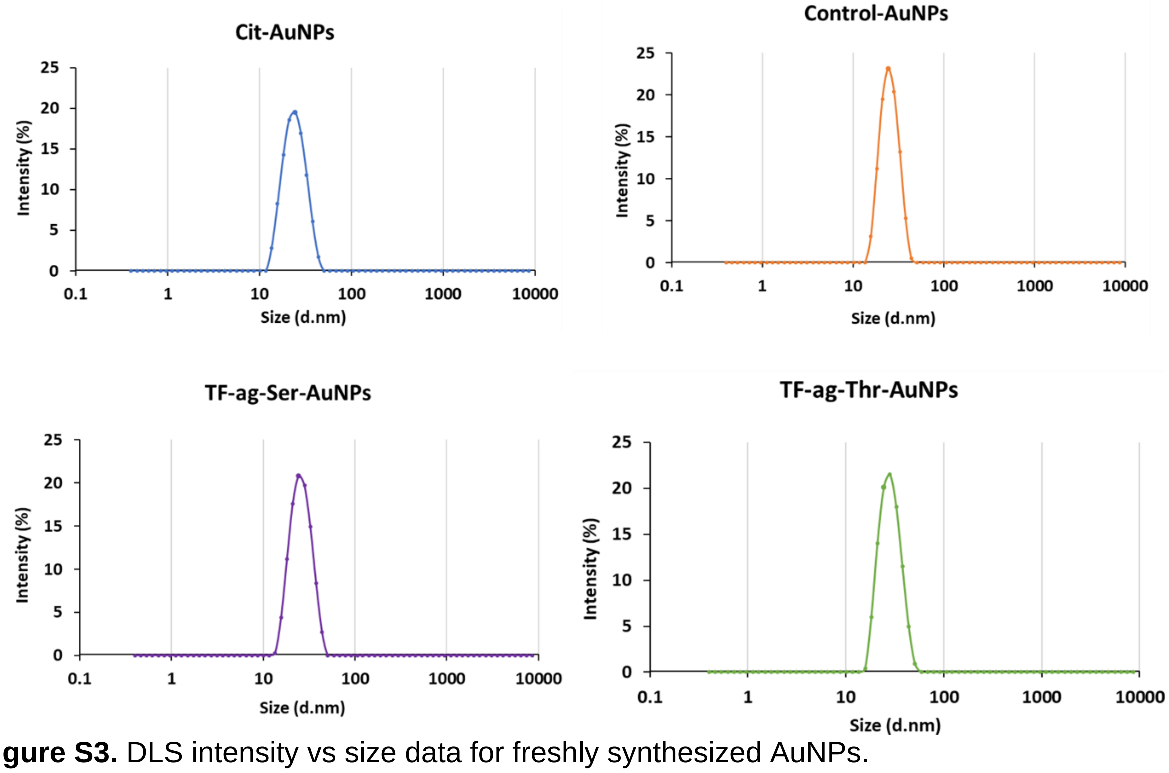

Figure S3. DLS intensity vs size data for freshly synthesized AuNPs.

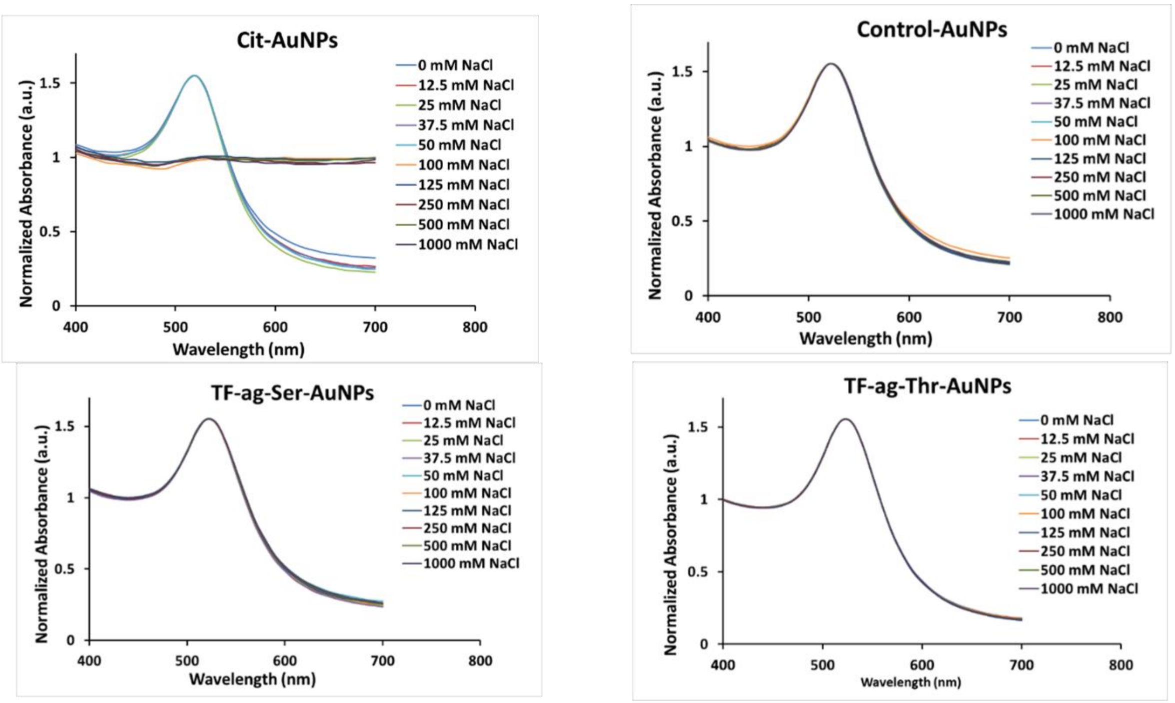

Figure S4. Stability of citate stabilized AuNPs vs. conjugated AuNPs in high salt.

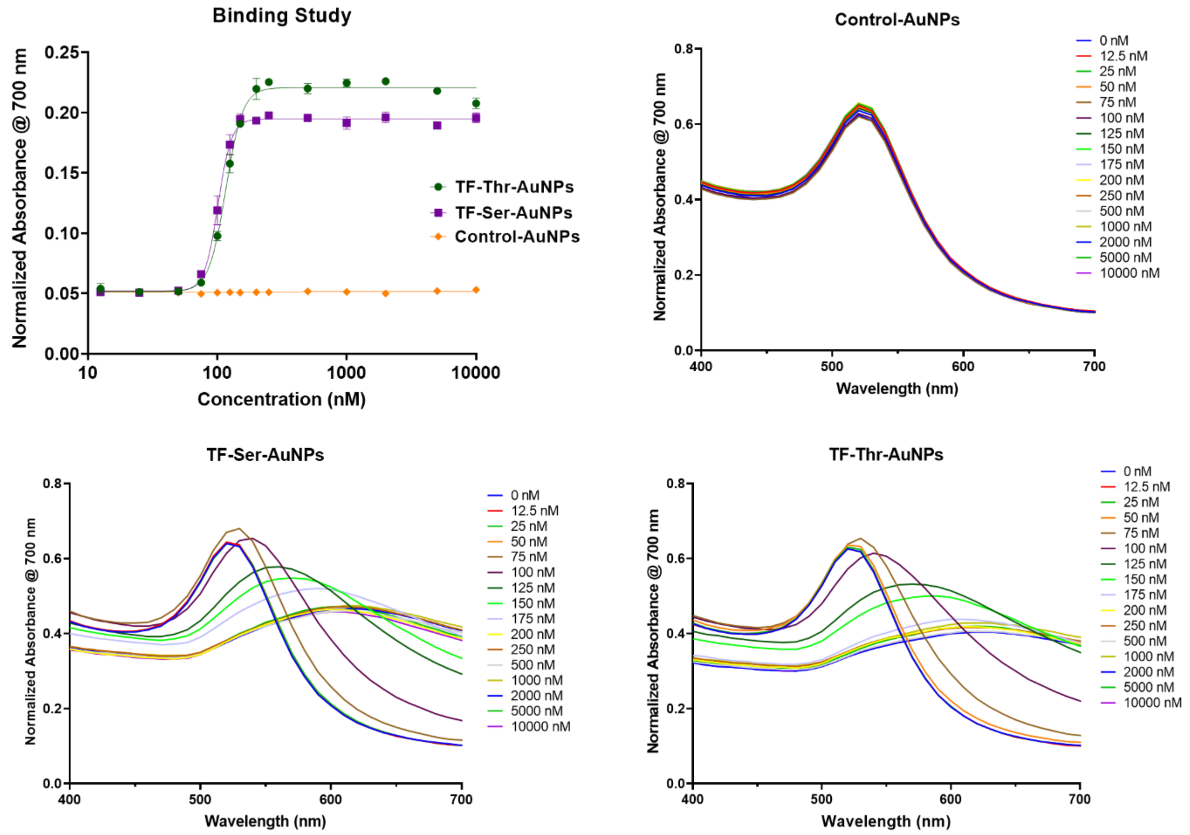

Figure S5. Aggregation of AuNPs by Gal-3 as observed by UV/Vis spectroscopy DLS. Upper left: Absorbance at 700 nm (indicator of aggregation) plotted vs increasing concentrations of Gal-3. As can be seen, control AuNPs were inert to Gal-3 addition while both TF-Ser and TF-Thr-conjugated AuNPs aggregated rapidly with addition of Gal-3.

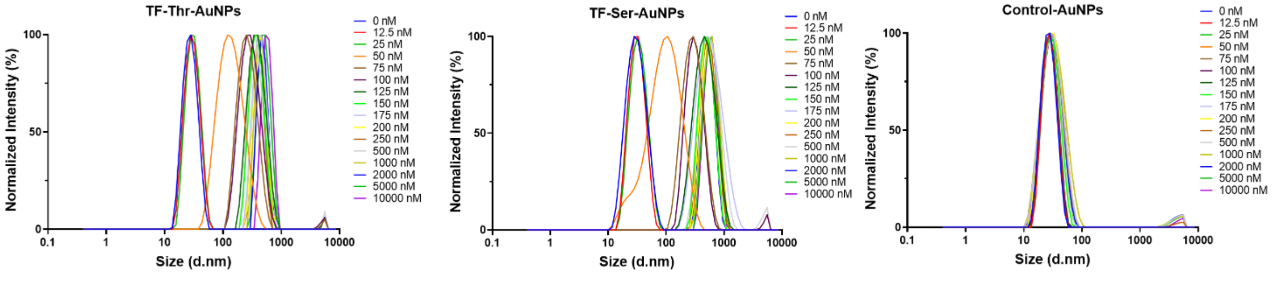

Figure S6. Aggregation of AuNPs by Gal-3 as observed by DLS spectroscopy DLS. As can be seen, control AuNPs were inert to Gal-3 addition while both TF-Ser and TF-Thr-conjugated AuNPs aggregated rapidly with addition of Gal-3.

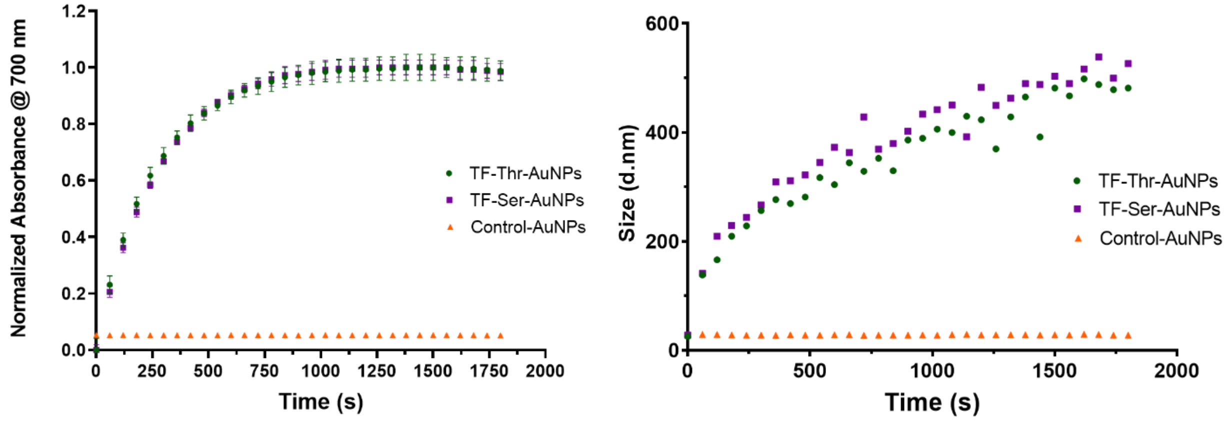

Figure S7. Kinetics of the aggregation phenomena from data in Figure S5 (left panel, absorbance at 700nm; right panel, DLS size measurements. See experimental description for Kd values).

Synthesis of iso-Lipoic Acid

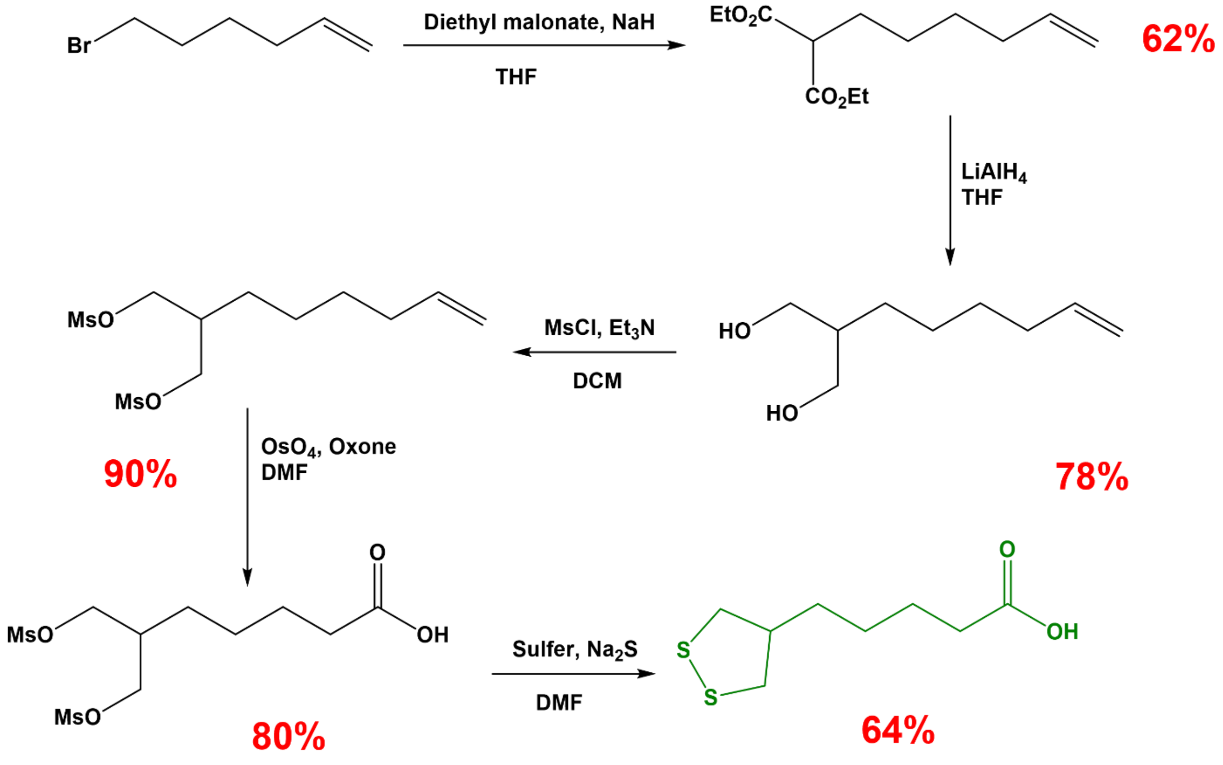

(Although published in 2010 by Tucker, et al., the synthetic methods we used are included here since it has not been reported anywhere else. Some slight modifications were made; see original publication for details)

Diethyl 2-(Hex-5-enyl)malonate

Diethyl malonate in THF (50 mL; 10.62 mL, 70.0 mmol) was very slowly added to a stirred suspension of sodium hydride in THF (150 mL, 2.80 g of a 60% w/w mixture in mineral oil, 70.0 mmol) over 45 min at 0°C. The resulting colorless reaction mixture was stirred for another 15 min at 0°C, then at room temperature for 45 min. The reaction mixture was again cooled down to 0°C, and a diluted solution of 6-bromo-1-hexene (8.50 mL, 63.6 mmol) in THF (50 mL) was added slowly over a period of 15 min. The reaction mixture was subsequently refluxed at 70°C for 18 h. The reaction was monitored by LC-MS, and upon completion was cooled to room temperature and quenched by the addition of 100 mL of cold water. Another 200 mL of water was added and the aqueous solution was extracted with ethyl acetate (3 × 100 mL). The organic layers were combined, washed three times with brine (3 × 100 mL), dried with MgSO4, and solvent was removed. The crude product was then purified by flash chromatography using hexane and ethyl acetate as mobile phase with a gradient of 0%-15% ethyl acetate for 27 min to afford the pure product as a colorless oil (10.5 g, 68%); **^{1}H NMR (400 MHz, $CDCl_3$)** δ 5.80 (ddtd, *J* = 17.0, 10.2, 6.7, 1.2 Hz, 1H, CH_2═C**H**), 5.06 – 4.90 (m, 2H, C**H_2**═CH), 4.21 (qt, *J* = 7.1, 1.0 Hz, 4H, OCH_2CH_3), 3.32 (td, *J* = 7.6, 1.0 Hz, 1H, CH_2CH), 2.06 (tdt, *J* = 8.0, 6.7, 1.4 Hz, 2H, CH_2), 1.91 (dddd, *J* = 8.8, 7.5, 5.9, 1.2 Hz, 2H, CH_2), 1.53 – 1.30 (m, 4H, -CH_2-CH_2), 1.28 (td, *J* = 7.1, 1.1 Hz, 6H, OCH_2CH_3). **^{13}C NMR (101 MHz, $CDCl_3$)** δ 169.54 (C), 138.54 (CH), 114.56 (CH_2), 61.26 (CH_2), 52.01 (CH), 33.38 (CH_2), 28.57 (CH_2), 28.44 (CH_2), 26.75 (CH_2), 14.07 (CH_3); m/z (ESI) 265 ([M + Na]+, 100%), 243.3 (4, [M + H]+).

2-(Hex-5-enyl)propane-1,3-diol

In a two-neck round bottom flask, lithium aluminum hydride (3.29 g, 87 mmol) was suspended in THF (200 mL) and cooled to 0°C under nitrogen gas. A solution of the above-prepared diethyl ester (10.49 g, 43.3mmol) pre-dissolved in THF (75 mL) was slowly added to this suspension over 15 min. The reaction mixture was allowed to stir for 45 min at 0 °C followed by warming to room temperature and stirring for another 6 h. THF (100 ml) was added and the solution was cooled to 0 °C. Cold water (10 mL) was added slowly followed by addition of aqueous sodium hydroxide solution (2 M; 10 mL). The reaction mixture was then stirred vigorously at room temperature for 12 h. The resulting reaction mixture was then filtered through Celite, and washed with ethyl acetate (100 mL). The filtrate was concentrated under reduced pressure using yielding the crude product which was purified by flash chromatography using Hexane and ethyl acetate to give the title compound as a colorless oil (5.35 g, 78%). **^{1}H NMR (400 MHz, $CDCl_3$)** δ 5.82 (ddt, *J* = 16.9, 10.1, 6.7 Hz, 1H, CH_2═C**H**), 5.06 – 4.92 (m, 2H, C**H_2**═CH), 3.83 (dd, *J* = 10.6, 3.8 Hz, 2H, C**H_2**OH), 3.67 (dd, *J* = 10.6, 7.6 Hz, 2H, C**H_2**OH), 2.46 (s, 2H, CH_2O**H**), 2.13 – 2.02 (m, 2H, C**H_2**), 1.85 – 1.72 (m, 1H, C**H**), 1.48 – 1.31 (m, 4H, -C**H_2**-C**H_2**-), 1.31 – 1.21 (m, 2H, -CH-C**H_2**). **^{13}C NMR (101 MHz, $CDCl_3$)** δ 138.84 (**C**H), 114.44 (**C**H_2), 66.61(**C**H_2), 41.95 (**C**H), 33.62 (**C**H_2), 29.08 (CH_2), 27.54 (**C**H_2), 26.65 (**C**H_2)); m/z (ESI) 223.3, 181 [M + Na]+).

Methanesulfonic Acid 2-Methanesulfonyloxymethyloct-7-enyl Ester

To a stirred solution of diol (4.11 g, 26 mmol in dichloromethane (250 mL), was slowly added triethylamine (18.12 mL, 130 mmol) at 0°C under argon. Methanesulfonyl chloride (8.10 mL, 104 mmol) was added slowly over 15 min. The reaction was stirred for another 15 min at 0 °C and then at room temperature overnight. The reaction was quenched with cold water (250 mL) and then extracted with Dichloromethane (3 × 125 mL). The organic layer was washed with brine

solution three times (3 x 300 mL), dried with magnesium sulfate ($MgSO_4$), filtered, and solvent was removed to yield the crude product as yellow oil. Purification by flash chromatography using Hexane and Ethyl acetate as mobile phase yielded the final compound as a yellowish oil (7.37 g, 90%); **^{1}H NMR (400 MHz, $CDCl_3$)** δ 5.80 (ddt, *J* = 16.9, 10.2, 6.7 Hz, 1H, CH_2═C**H**), 5.12 – 4.80 (m, 2H, C**H_2**═CH), 4.30 (dd, *J* = 10.0, 4.2 Hz, 2H, CHC**H_2**O), 4.21 (dd, *J* = 10.0, 6.4 Hz, 2H, CHC**H_2**O), 3.06 (s, 6H, SO_2**Me**), 2.18 (dd, *J* = 7.9, 3.6 Hz, 1H, C**H**), 2.08 (q, *J* = 6.5 Hz, 2H, C**H_2**), 1.50 – 1.38 (m, 6H, C**H_2**), **^{13}C NMR (101 MHz, $CDCl_3$)** δ 138.35 (**C**H), 114.85 (**C**H_2), 68.18 (**C**H_2), 38.22 (**C**H), 37.36 (**C**H_3), 33.40 (**C**H_2), 28.67 (**C**H_2), 26.89 (**C**H_2), 25.97 (**C**H_2) ; m/z (ESI) 337.3 ([M + Na]+

7-((methylsulfonyl)oxy)-6-(((methylsulfonyl)oxy)methyl)heptanoic acid

Osmium tetroxide (1.24 mL of a 2.5% w/w solution in tBuOH, 0.099 mmol) was added to a stirred solution of the olefin (3.10 g, 9.86 mmol) in DMF (65 mL), and the resulting mixture was stirred for 5 min. Methanesulfonic acid 2-methanesulfonyloxymethyloct-7-enyl ester (2.088 g, 6.1 mmol) was dissolved in dry DMF (50 mL). Osmium tetroxide (0.766 mL of a 2.5% w/w solution in tBuOH, 0.061 mmol) was added to the reaction vessel and stirred for 5 min. After that oxone (15 g, 24.4 mmol) was added to the reaction mixture in three parts over 15 min and allowed to stir for 3.5 h at room temperature. Sodium sulfite (13.84 g) was then added in two portions and stirred vigoriously for another 2 h. The reaction was then quenched by adding water (100 mL), aqueous hydrochloric acid (2 M, 40 mL). The reaction mixture was then filtered and ethyl acetate:ether (1:1, 200 mL) was added and extracted with another 150 mL of ethyl acetate:ether (1:1, 150 mL) mixture. The organic layer was then dried, evaporated and purified by flash chromatography to yield the title compound as a white solid (1.75 g, 80%). **^{1}H NMR (400 MHz, $CDCl_3$)** δ 10.32 (s, 1H, -COO**H**), 4.22 (dd, *J* = 10.1, 4.3 Hz, 2H, CHC**H_2**O), 4.14 (dd, *J* = 10.1, 6.4 Hz, 2H, CHC**H_2**O), 2.98 (s, 6H, -SO_2**Me**), 2.32 (t, *J* = 7.2 Hz, 2H, C**H**$_2$), 2.11 (dt, *J* = 10.9, 6.6 Hz, 1H, C**H**), 1.59 (d, *J* = 6.7 Hz, 2H, -C**H_2**), 1.39 (q, *J* = 3.6 Hz, 4H, -**C**H_2-CH_2). **^{13}C NMR (101 MHz, $CDCl_3$)** δ 179.23 (-**C**OOH), 68.13 (**C**HCH_2O), 38.01 (-**C**H_2-COOH), 37.32 (-SO_2**CH_3)** , 33.57 (CH**)**, 26.68 (**C**H_2**)**, 25.86 (**C**H_2**)**, 24.37 (**C**H_2**)**.

Isolipoic Acid

To a stirring solution of 7-((methylsulfonyl)oxy)-6-(((methylsulfonyl)oxy)methyl)heptanoic acid (1.75 g, 4.85 mmol) in DMF (100 mL), was slowly added sulfur (155 mg, 4.85 mmol) followed by addition of sodium sulfide hydrate (1.17 g, 4.85 mmol). After 10 mins, the reaction mixture was heated at 85°C for 6 h. The reaction mixture turned yellow once it completed. The reaction mixture was then allowed to cool down and DMF was removed by rotary evaporator with bath temperature set at 60°C. To the dried crude reaction mixture, cold water (100 mL) and diethyl ether (100 mL) were added. The water layer was extracted with cold diethyl ether (2 × 100 mL), washed with brine solution (2 ×100 mL) and dried with ($MgSO_4$). The organic extract was then dried by rotary evaporator. The crude reaction product was then purified by flash chromatography using DCM and methanol with gradient up to 10% of methanol for 25 min. The useful fractions were identified by LC-MS and combined to yield the purified product as yellow solid (0.641 g, 64%). **1H NMR (400 MHz, Chloroform-d)** δ 3.27 (dd, J = 11.0, 6.6 Hz, 2H, C**H_2**), 2.81 (dd, J = 11.1, 6.7 Hz, 2H, C**H_2**), 2.56 (p, J = 6.8 Hz, 1H, CH_2C**H**(CH_2S)$_2$), 2.39 (t, J = 7.3 Hz, 2H, C**H_2**), 1.68 (p, J = 7.4 Hz, 2H, C**H_2**), 1.55 (dt, J = 9.5, 6.1 Hz, 2H, C**H_2**), 1.45 (dddd, J = 14.3, 9.3, 6.6, 2.1 Hz, 2H, C**H_2**). δC(100 MHz; CDCl3) 179.7 (C), 47.5 (CH), 43.9 (CH2), 33.8 (CH2), 33.4 (CH2), 27.9 (CH2), 24.5 (CH2); m/z (ESI) 205.1 ([M − H]$^-$, 27%), 171 (100)

Procedure for the synthesis of Control ligand (LA-PEG-OH)

In a dry 20 mL vial, EDC•HCl (0.192 g, 1 mmol) and 1-hydroxybenzotriazole hydrate (0.153 g, 1 mmol) were dissolved in dry DCM (5 mL) at 0°C. After 10 min, Isolipoic acid (0.103 g, 0.5 mmol) dissolved in DCM (2 mL) was added in one batch. The reaction mixture was stirred for 45 min at 0°C. After that, Amino-PEG_7-alcohol **10** (0.163 g, 0.5 mmol) was added to the reaction mixture followed by addition of DIPEA (35 µL, 0.2 mmol). The reaction was then stirred for 12 h at room temperature. The reaction was monitored by LC-MS for completion. The reaction mixture was then absorbed into silica gel and purified by flash chromatography using DCM and methanol with a gradient of up to 10% methanol for 30 min. The useful fractions were identified by LC-MS and combined and subjected to another purification step via HPLC using water and acetonitrile as the mobile phase to yield the title compound **11** as yellowish sticky solid (0.22 g, 86%).

^{1}H NMR (500 MHz, $CDCl_3$) δ 6.24 (t, *J* = 5.6 Hz, 1H, -N**H**), 3.70 – 3.63 (m, 2H, HO-C**H₂**-CH₂-), 3.62 – 3.51 (m, 22H, [-(O-C**H₂**-C**H₂**-O-)₅-C**H₂**-CH₂-NH-), 3.51 – 3.46 (m, 2H, HO-CH₂-C**H₂**-), 3.37 (q, *J* = 5.3 Hz, 2H, -O-CH₂-C**H₂**-NH-), 3.18 (dd, *J* = 11.1, 6.6 Hz, 2H, -C**H₂**-S-S-C**H₂**-), 2.91 (s, 1H, **H**O-CH₂-CH₂-), 2.72 (dd, *J* = 11.0, 6.7 Hz, 2H, -C**H₂**-S-S-C**H₂**-), 2.47 (hept, *J* = 6.8 Hz, 1H, -CH₂C**H**(CH₂S)₂), 2.13 (t, *J* = 7.5 Hz, 2H, -CO-C**H₂**-), 1.59 (p, *J* = 7.5 Hz, 2H, -CO-CH₂-C**H₂**-), 1.46 (dt, *J* = 9.5, 7.1 Hz, 2H, -CH₂-CH₂-C**H₂**-), 1.38 – 1.28 (m, 2H, -CH₂-C**H₂**-CH-).**^{13}C NMR (126 MHz, $CDCl_3$)** δ 172.82 (-CH₂-**C**O-NH-), 77.38 (HO-**C**H₂-CH₂-), (77.13, 76.87, 72.51, 70.57, 70.53, 70.51, 70.49, 70.47, 70.29, 70.17, 69.87 [-(O-**C**H₂-**C**H₂-O-)₅-**C**H₂-CH₂-NH-], 61.62 (HO-CH₂-**C**H₂-), 47.64 (-O-CH₂-**C**H₂-NH-), 43.93 (-**C**H₂-S-S-**C**H₂-), 39.14 (-CH₂**C**H(CH₂S)₂), 36.22 (-CO-**C**H₂-), 33.51 (-CH₂-**C**H₂-CH-), 28.16 (-CH₂-CH₂-**C**H₂-), 25.56 (-CO-CH₂-**C**H₂-). HRMS: Measured MH+ = m/z 514.2497, calculated MH+ = 514.2503

Procedure for the synthesis of ILA-PEG_6-NH_2 (3)

EDC•HCl (0.192 g, 1 mmol) and 1-hydroxybenzotriazole hydrate (0.153 g, 1 mmol) in dry DCM (5 mL) was stirred at 0°C in a 20 mL glass vial. After 5 min, Isolipoic acid (0.103 g, 0.5 mmol) in DCM (2 mL) was added to the reaction mixture and stirred 45 min at room temperature. t-Boc-N-amido-PEG6-amine (**1**) (0.212 g, 0.5 mmol) in dry DCM (2 mL) and DIPEA (45 µL, 0.32 mmol) were added sequentially to the reaction mixture. The reaction was stirred for 12 h and monitored by LC-MS for reaction completion. The crude reaction mixture was then incorporated into silica gel and subjected to purification by flash chromatography using DCM and methanol (9:1) as eluent for 35 min. The pure fractions were combined and purified again by HPLC to give the **ILA-PEG_6-Tboc (2)** as sticky yellowish solid (0.275 g, 90%).

50% Trifluoroacetic acid in DCM (2 mL) was added to the **ILA-PEG_6-Tboc** (0.275, 0.5 mmol) conjugate in a 20 mL glass vial. The reaction was stirred for 6 h at room temperature. TFA was removed under nitrogen gas flow, incorporated into silica gel and purified by flash chromatography using DCM and methanol as mobile phase with a gradient of up to 10% methanol. The pure fractions were identified, combined and re-purified by HPLC using water and acetonitrile as mobile phase to give the **ILA-PEG_6-NH_2** linker (**3**) as a yellowish sticky solid (0.21 g, 91%).

ILA-PEG_6-Tboc Conjugate (2)

^{1}H NMR (500 MHz, $CDCl_3$) δ 6.23 (s, 1H, -CH₂-N**H**-CO-), 5.06 (s, 1H, -CH₂-N**H**-COO-), 3.71 – 3.60 (m, 20H, -(O-C**H₂**-C**H₂**-O-)₅), 3.56 (dt, *J* = 10.5, 5.0 Hz, 4H, -O-C**H₂**-C**H₂**-NH-), 3.47 (q, *J* = 4.9 Hz, 2H, -C**H₂**-NH-COO-), 3.32 (t, *J* = 5.1 Hz, 2H, -C**H₂**-CH₂-NH-COO-), 3.26 (dd, *J* = 11.0, 6.6 Hz, 2H, -C**H₂**-S-S-C**H₂**-), 2.81 (dd, *J* = 11.0, 6.7 Hz, 2H, -C**H₂**-S-S-C**H₂**-), 2.55 (hept, *J* = 6.8 Hz, 1H, -CH₂C**H**(CH₂S)₂), 2.22 (t, *J* = 7.5 Hz, 2H, -CO-C**H₂**-), 1.72 – 1.64 (m, 2H, -CO-CH₂-C**H₂**-), 1.58 – 1.50 (m, 2H, -CH₂-CH₂-C**H₂**-), 1.46 (s, 9H, -C-(C**H₃**)₃), 1.44 – 1.39 (m, 2H, -CH₂-C**H₂**-

CH-). **^{13}C NMR (126 MHz, $CDCl_3$)** δ 172.79 (-CH_2-NH-**C**O-), 156.01 (-CH_2-NH-**C**OO-), 79.16 -**C**-$(CH_3)_3$, [(77.31, 77.06, 76.80, 70.56, 70.54, 70.51, 70.24, 70.22, 70.20, 69.94), [1]], 47.67 (-CH_2-NH-**C**O-), 43.95 (-**C**H_2-S-S-**C**H_2-), 40.36 (-CH_2**C**H(CH_2S)$_2$), 39.15 (-**C**H_2-NH-COO-), 36.26 (-CO-**C**H_2-), 33.54 (-CH_2-**C**H_2-CH-), 28.44 {-C-(**C**H_3)$_3$}, 28.19 (-CH_2-CH_2-**C**H_2-), 25.58 (-CO-CH_2-**C**H_2-). HRMS: Measured MH+ = m/z 613.3189, calculated MH+ = 613.3187

ILA-PEG$_6$-NH$_2$ Conjugate (3)

^{1}H NMR (500 MHz, $CDCl_3$) δ 7.83 (s, 1H-CH_2-N**H**-CO-), 3.90 – 3.84 (m, 2H, -O-C**H₂**CH_2-NH_2), 3.79 – 3.58 (m, 24H, -CO-NH-C**H₂**-C**H₂**-(O-C**H₂**-C**H₂**-O-)$_5$), 3.45 (q, *J* = 5.2 Hz, 2H, -O-CH_2C**H₂**-NH_2), 3.27 (dd, *J* = 11.0, 6.6 Hz, 2H, -C**H₂**-S-S-C**H₂**-), 3.21 (p, *J* = 5.4 Hz, 2H, -O-CH_2-CH_2-N**H₂**), 2.81 (dd, *J* = 11.0, 6.9 Hz, 2H, -C**H₂**-S-S-C**H₂**-), 2.55 (hept, *J* = 6.8 Hz, 1H, -CH_2C**H**(CH_2S)$_2$), 2.28 (t, *J* = 7.5 Hz, 2H, -CO-C**H₂**-), 1.67 (p, *J* = 7.6 Hz, 2H, -CO-CH_2-C**H₂**-), 1.58 – 1.50 (m, 2H, -CH_2-CH_2-C**H₂**-), 1.47 – 1.37 (m, 2H, -CH_2-C**H₂**-CH-).**^{13}C NMR (126 MHz, $CDCl_3$)** δ 173.99 (-CH_2-NH-**C**O-), [70.34, 70.29, 70.19, 69.93, 69.88, 69.79, 69.74, 69.55, 69.49, 69.23, {-CO-NH-CH_2-CH_2-(O-CH_2-CH_2-O-)$_5$-}], 67.11 (-O-**C**$H_2$$CH_2$-$NH_2$), 47.82 (-O-$CH_2$**C**$H_2$-$NH_2$), 43.97 (-$CH_2$-S-S-$CH_2$-), 40.16 (-$CH_2$**C**H($CH_2$S)$_2$), 39.04 (-CO-NH-**C**$H_2$-), 35.88 (-CO-**C**$H_2$-), 33.51 (-$CH_2$-**C**$H_2$-CH-), 28.19 (-$CH_2$-$CH_2$-**C**$H_2$-), 25.73 (-CO-$CH_2$-**C**$H_2$-). HRMS: Measured MH+ = m/z 513.2656, calculated MH+ = 513.2663

General Procedure for the synthesis of ILA-PEG$_6$-TF-Ser/Thr-NH-Fmoc conjugates

To a stirring solution of TF-Ser/Thr amino acid (0.144 g, 0.75 mmol) in DCM (7 mL) were added EDC.HCl (0.144 g, 0.75 mmol) and 1-hydroxybenzotriazole hydrate (0.115 g, 0.75 mmol) at 0°C. After 45 mins, **ILA-PEG$_6$-NH$_2$ (3)** (0.144 g, 0.75 mmol) in DCM (2 mL) and DIPEA (5 µL, 0.3 mmol) were added to the reaction mixture and stirred for 12 h at room temperature. The reaction was monitored by LC-MS, once completed the reaction mix was absorbed into silica and purified by flash chromatography using DCM and methanol (9:1) as mobile phase for 30 min. Pure fractions were identified, combined and purified again by HPLC using water and acetonitrile as eluent to yield the conjugate as white solid.

ILA-PEG$_6$-TF-Ser-NH-Fmoc conjugate (4)

^{1}H NMR (500 MHz, $CDCl_3$) δ 7.69 (d, *J* = 7.5 Hz, 2H), 7.54 (d, *J* = 7.5 Hz, 2H), 7.33 (t, *J* = 7.5 Hz, 2H), 7.24 (t, *J* = 7.4 Hz, 2H), 6.42 (s, 1H), 5.31 (s, 1H), 5.27 (d, *J* = 3.3 Hz, 1H), 5.00 (t, *J* = 9.1 Hz, 1H), 4.87 (d, *J* = 10.9 Hz, 1H), 4.74 (d, *J* = 3.6 Hz, 1H), 4.43 (t, *J* = 9.8 Hz, 1H), 4.36 (s, 1H), 4.31 (d, *J* = 7.0 Hz, 2H), 4.16 (t, *J* = 7.0 Hz, 1H), 4.05 – 4.00 (m, 3H), 3.99 – 3.94 (m, 1H), 3.91 (dd, *J* = 11.1, 6.7 Hz, 1H), 3.83 (s, 1H), 3.75 (s, 2H), 3.51 (d, *J* = 10.8 Hz, 22H), 3.44 (t, *J* = 5.2 Hz, 2H), 3.39 (s, 2H), 3.34 (t, *J* = 5.4 Hz, 2H), 3.14 (dd, *J* = 11.0, 6.6 Hz, 2H), 2.69 (dd, *J* = 11.1, 6.5 Hz, 2H), 2.44 (hept, *J* = 6.7 Hz, 1H), 2.12 (t, *J* = 7.5 Hz, 2H), 2.07 (s, 3H), 2.05 (s, 3H), 1.96 (d, *J* = 1.8 Hz, 9H), 1.90 (s, 3H), 1.88 (s, 3H), 1.55 (p, *J* = 7.5 Hz, 2H), 1.45 – 1.37 (m, 2H), 1.34 – 1.24 (m, 2H). **^{13}C NMR (126 MHz, $CDCl_3$)** δ 173.93, 170.64, 170.50, 170.44, 170.27, 170.14, 169.66, 160.56, 160.26, 143.74, 141.29, 141.27, 127.83, 127.17, 125.11, 120.03, 116.88, 114.58, 100.97, 98.69, 77.27, 77.02, 76.77, 70.82, 70.34, 70.10, 68.72, 67.73, 67.32, 66.91, 62.73, 60.84, 49.11, 47.54, 47.01, 43.89, 39.33, 39.06, 36.11, 33.42, 28.04, 25.61, 22.77, 20.80, 20.77, 20.74, 20.64, 20.60. HRMS: Measured MH+ = m/z 1439.5628, calculated MH+ = 1439.5620

ILA-PEG$_6$-TF-Thr-NH-Fmoc conjugate (5)

^{1}H NMR (500 MHz, MeOD) δ 8.16 (t, *J* = 5.3 Hz, 1H), 7.85 (d, *J* = 7.5 Hz, 2H), 7.71 (dd, *J* = 7.7, 3.8 Hz, 2H), 7.47 – 7.32 (m, 4H), 7.18 (d, *J* = 9.2 Hz, 1H), 5.39 (dd, *J* = 16.8, 3.2 Hz, 2H), 5.07 – 4.97 (m, 2H), 4.86 (d, *J* = 3.7 Hz, 1H), 4.70 (d, *J* = 7.2 Hz, 1H), 4.61 (dd, *J* = 10.8, 6.4 Hz, 1H),

4.52 (dd, J = 10.8, 6.2 Hz, 1H), 4.37 (td, J = 10.6, 3.6 Hz, 1H), 4.30 (t, J = 6.2 Hz, 1H), 4.26 – 4.20 (m, 3H), 4.20 – 4.11 (m, 3H), 4.09 – 3.93 (m, 3H), 3.66 – 3.57 (m, 22H), 3.53 (dt, J = 9.6, 5.2 Hz, 4H), 3.36 (t, J = 5.4 Hz, 2H), 3.25 (dd, J = 11.0, 6.6 Hz, 2H), 2.79 (dd, J = 11.0, 6.5 Hz, 2H), 2.53 (hept, J = 6.7 Hz, 1H), 2.22 (t, J = 7.4 Hz, 2H), 2.15 (s, 3H), 2.13 (s, 3H), 2.10 – 2.01 (m, 12H), 1.95 (s, 3H), 1.64 (p, J = 7.5 Hz, 2H), 1.56 – 1.48 (m, 2H), 1.41 (qd, J = 8.2, 5.4 Hz, 2H), 1.26 (d, J = 6.2 Hz, 3H).**^{13}C NMR (126 MHz, MeOD)** δ 174.72, 171.70, 171.02, 170.94, 170.84, 170.66, 170.63, 170.09, 169.70, 157.49, 143.94, 143.76, 141.31, 127.50, 126.86, 124.76, 124.63, 119.68, 119.64, 101.06, 99.63, 78.46, 74.61, 72.78, 70.82, 70.33, 70.17, 70.16, 70.14, 70.11, 70.10, 69.87, 69.85, 69.20, 68.86, 68.76, 67.62, 67.14, 66.31, 62.83, 60.86, 58.95, 43.38, 38.96, 35.38, 33.14, 27.60, 25.53, 22.06, 19.50, 19.38, 19.32, 19.29, 19.08, 19.07, 17.85. HRMS: Measured MH+ = m/z 1453.5787, calculated MH+ = 1453.5776

General Procedure for the synthesis of ILA-PEG$_6$-TF-Ser/Thr-NH-Ac conjugate

The Fmoc group of ILA-PEG$_6$-TF-Ser/Thr-NH-Fmoc conjugates **4** and **5** were deprotected by using Piperidine. Briefly, to a solution of ILA-PEG$_6$-TF-Ser/Thr-NH-Fmoc conjugates in DMF (1 mL) was added 10% piperidine in DMF (1 mL) at room temperature. The reaction is stirred for 30 mins and reaction was monitor by LC-MS. After reaction completion, DMF was removed from the reaction mixture by a Genevac centrifugal evaporator. The dried reaction product was dissolved in dry pyridine (1 mL) and excess acetic anhydride (0.5 mL) was added at 0°C. The reaction mixture was stirred for 12 h. The solvent and reagent were removed under reduced pressure by Genevac centrifugal evaporator. The resulting crude product was first purified by flash chromatography by eluting with DCM and methanol with gradient set up to 10% of methanol for 30 min. Pure fractions ware identified by LC-MS, combined and further purified by HPLC using water and acetonitrile as mobile phase to give the title compound as white solid.

ILA-PEG$_6$-TF-Ser-NH-Ac conjugate (6)

^{1}H NMR (500 MHz, $CDCl_3$) δ 7.63 (s, 1H), 6.85 (s, 1H), 6.69 (s, 1H), 6.53 (s, 1H), 5.38 (dd, J = 9.5, 3.4 Hz, 2H), 5.11 (dd, J = 10.5, 7.8 Hz, 1H), 4.97 (dd, J = 10.5, 3.6 Hz, 1H), 4.84 (d, J = 3.7 Hz, 1H), 4.71 (dt, J = 8.3, 4.5 Hz, 1H), 4.65 (s, 1H), 4.54 (td, J = 10.1, 3.6 Hz, 1H), 4.23 – 4.10 (m, 3H), 4.07 (t, J = 6.2 Hz, 1H), 3.98 (dd, J = 11.3, 7.2 Hz, 1H), 3.92 (s, 4H), 3.94 – 3.85 (m, 1H), 3.71 – 3.63 (m, 24H), 3.51 – 3.41 (m, J = 5.1, 4.6 Hz, 2H), 3.40 (s, 3H), 3.26 (dd, J = 11.0, 6.6 Hz, 2H), 2.81 (dd, J = 11.0, 6.6 Hz, 2H), 2.78 (s, 1H), 2.56 (p, J = 6.8 Hz, 1H), 2.26 (t, J = 7.4 Hz, 2H), 2.19 (s, 3H), 2.15 (s, 3H), 2.09 (d, J = 5.2 Hz, 12H), 2.06 (s, 3H), 1.99 (s, 3H), 1.68 (p, J = 7.5 Hz, 2H), 1.58 – 1.50 (m, 2H), 1.42 (ddt, J = 15.7, 11.3, 5.8 Hz, 2H). **^{13}C NMR (126 MHz, $CDCl_3$)** δ 173.64, 170.71, 170.68, 170.65, 170.49, 170.45, 170.23, 170.19, 170.01, 169.54, 101.06, 98.89, 77.28, 77.02, 76.77, 73.58, 70.91, 70.73, 70.50, 70.37, 70.05, 69.84, 69.06, 68.68, 67.78, 66.77, 62.74, 61.05, 60.93, 52.88, 48.71, 47.64, 43.93, 39.55, 39.32, 36.17, 36.12, 33.49, 28.13, 25.67, 23.16, 23.09, 20.81, 20.76, 20.72, 20.70, 20.64, 20.59, 20.35. Measured MH+ = m/z 1259.5, calculated MH+ = 1258.5.

ILA-PEG$_6$-TF-Thr-NH-Ac conjugate (7)

^{1}H NMR (400 MHz, $CDCl_3$) δ 7.96 (s, 1H), 7.04 (s, 2H), 6.71 (s, 1H), 6.28 (s, 4H), 5.39 (t, J = 3.9 Hz, 2H), 5.16 – 5.04 (m, 1H), 4.96 (dd, J = 10.5, 3.4 Hz, 1H), 4.86 (d, J = 3.7 Hz, 1H), 4.67 (d, J = 7.7 Hz, 1H), 4.64 – 4.57 (m, 1H), 4.51 (td, J = 10.2, 3.7 Hz, 1H), 4.28 – 4.21 (m, 1H), 4.21 (d, J = 4.7 Hz, 1H), 4.21 – 4.07 (m, 2H), 4.05 – 3.89 (m, 3H),

3.72 – 3.56 (m, 20H), 3.56 (t, *J* = 5.3 Hz, 5H), 3.47 (dd, *J* = 12.0, 6.8 Hz, 2H), 3.33 (s, 1H), 3.25 (dd, *J* = 11.0, 6.5 Hz, 2H), 2.81 (dd, *J* = 11.0, 6.6 Hz, 2H), 2.56 (h, *J* = 6.8 Hz, 1H), 2.26 (t, *J* = 7.5 Hz, 2H), 2.20 – 2.13 (m, 9H), 2.10 – 2.00 (m, 12H), 1.98 (s, 3H), 1.67 (p, *J* = 7.5 Hz, 2H), 1.53 (dt, *J* = 9.4, 6.3 Hz, 2H), 1.48 – 1.35 (m, 2H), 1.31 (d, *J* = 6.3 Hz, 3H). **^{13}C NMR (126 MHz, $CDCl_3$)** δ 172.17, 169.57, 169.26, 168.89, 168.66, 168.63, 168.56, 168.36, 168.27, 168.06, 167.59, 167.54, 99.12, 98.67, 75.39, 75.13, 74.88, 71.24, 68.85, 68.51, 68.20, 68.14, 67.90, 67.51, 66.79, 65.99, 64.95, 61.18, 58.94, 55.26, 47.11, 45.70, 45.67, 41.99, 37.60, 37.45, 37.38, 34.15, 31.52, 29.04, 26.15, 23.76, 21.06, 20.94, 18.91, 18.84, 18.82, 18.81, 18.75, 18.71, 18.67, 18.60, 18.58, 16.65. Low res MS: Measured MH+ = m/z 1273.6, calculated MH+ = 1272.5

General Procedure for the synthesis of ILA-PEG$_6$-TF-Ser/Thr final conjugate

In a dried 20 mL vial, ILA-PEG$_6$-TF-Ser/Thr-NH-Ac conjugates **6** or **7** was dissolved in dry methanol (1 mL). Sodium methoxide (NaOMe) (0.05 mM, 1 mL) was added to the reaction solution and allowed to stir for 30 mins. The completion of reaction was monitored by LC-MS. Once completed, the reaction was neutralized by adding few drops of acetic acid. The solvent was removed under reduced pressure, dissolved in water and acetonitrile (1:1) and finally purified by HPLC using water and acetonitrile as the mobile phase. The fractions were identified by LC-MS, combined and evaporated under reduced pressure in a Genevac centrifugal evaporator.

ILA-PEG$_6$-TF-Ser final conjugate (8)

^{1}H NMR (400 MHz, D_2O) δ 4.81 (d, *J* = 3.8 Hz, 1H), 4.51 (dd, *J* = 5.9, 4.2 Hz, 1H), 4.40 (d, *J* = 7.7 Hz, 1H), 4.25 (dd, *J* = 11.0, 3.8 Hz, 1H), 4.16 (d, *J* = 3.1 Hz, 1H), 3.98 – 3.81 (m, 4H), 3.78 – 3.70 (m, 1H), 3.67 (dq, *J* = 5.3, 3.2, 2.1 Hz, 4H), 3.65 – 3.58 (m, 22H), 3.54 (dt, *J* = 8.2, 4.9 Hz, 4H), 3.44 (dd, *J* = 10.0, 7.7 Hz, 1H), 3.33 (dt, *J* = 14.5, 5.3 Hz, 4H), 3.23 (dd, *J* = 11.1, 6.2 Hz, 2H), 2.80 (dd, *J* = 11.1, 6.9 Hz, 2H), 2.52 (h, *J* = 6.7 Hz, 1H), 2.20 (t, *J* = 7.3 Hz, 2H), 2.01 (s, 3H), 1.95 (s, 3H), 1.55 (p, *J* = 7.3 Hz, 2H), 1.49 – 1.39 (m, 2H), 1.32 (td, *J* = 7.8, 7.3, 5.0 Hz, 2H). HRMS: Measured MH+ = m/z 1007.4418, calculated MH+ = 1007.4411

ILA-PEG$_6$-TF-Thr final conjugate (9)

^{1}H NMR (500 MHz, D_2O) δ 4.73 (d, *J* = 3.8 Hz, 1H), 4.63 (s, 3H), 4.43 (t, *J* = 5.0 Hz, 1H), 4.32 (dd, *J* = 7.8, 2.1 Hz, 1H), 4.17 (dt, *J* = 11.1, 2.6 Hz, 1H), 4.08 (d, *J* = 3.1 Hz, 1H), 3.89 – 3.83 (m, 1H), 3.86 – 3.75 (m, 2H), 3.77 – 3.73 (m, 1H), 3.67 (q, *J* = 5.2 Hz, 1H), 3.64 – 3.56 (m, 4H), 3.56 – 3.49 (m, 22H), 3.52 – 3.40 (m, 4H), 3.36 (dd, *J* = 9.9, 7.8 Hz, 1H), 3.30 – 3.21 (m, 5H), 3.15 (dd, *J* = 11.1, 6.2 Hz, 1H), 2.72 (dd, *J* = 11.1, 6.9 Hz, 1H), 2.43 (hept, *J* = 6.8 Hz, 1H), 2.16 – 2.08 (m, 2H), 1.93 (d, *J* = 1.5 Hz, 3H), 1.88 (d, *J* = 1.3 Hz, 3H), 1.53 – 1.42 (m, 3H), 1.42 – 1.32 (m, 1H), 1.28 – 1.18 (m, 2H).**^{13}C NMR (126 MHz, D_2O)** δ 177.05, 174.67, 174.13, 171.56, 104.69, 98.87, 76.82, 75.15, 74.99, 72.46, 70.99, 70.52, 69.62, 69.59, 69.56, 69.43, 69.37, 68.84, 68.77, 68.68, 68.56, 61.20, 61.01, 57.94, 48.52, 47.54, 43.21, 39.01, 38.85, 35.46, 32.01, 26.90, 25.24, 22.25, 21.75, 18.12. HRMS: Measured MH+ = m/z 1021.4568, calculated MH+ = 1021.4567

Diethyl 2-(Hex-5-enyl)malonate

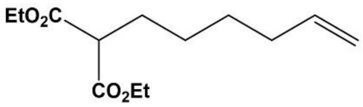

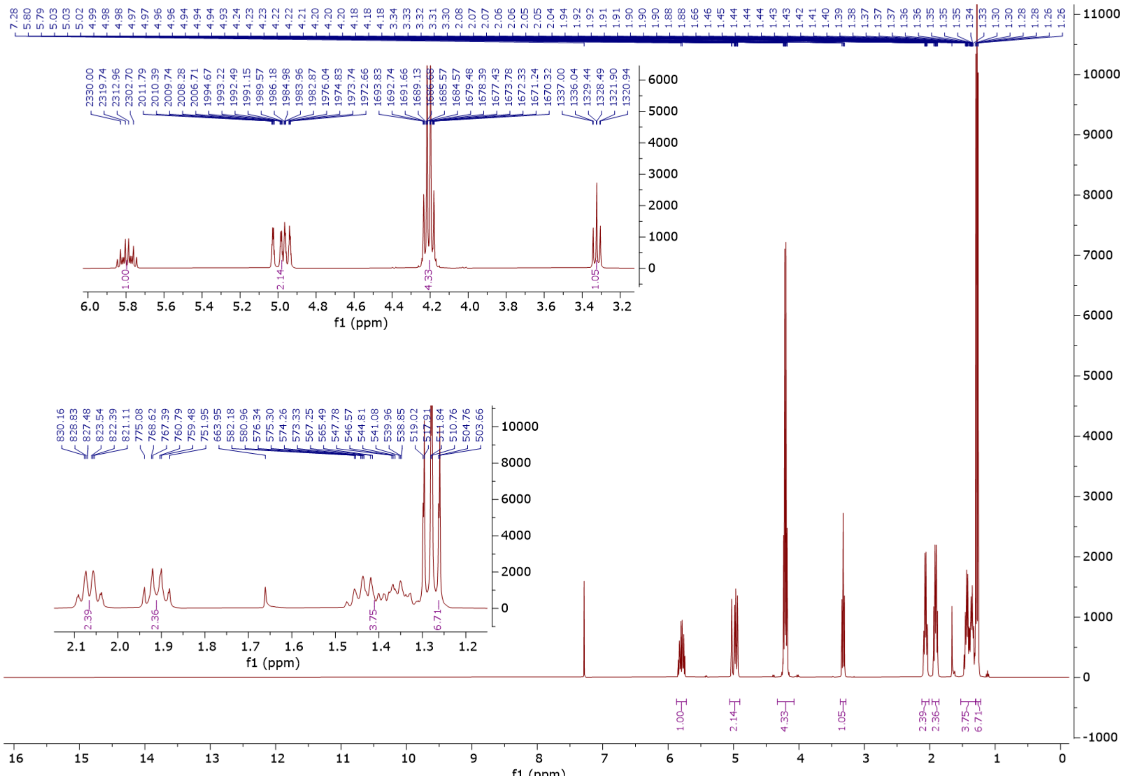

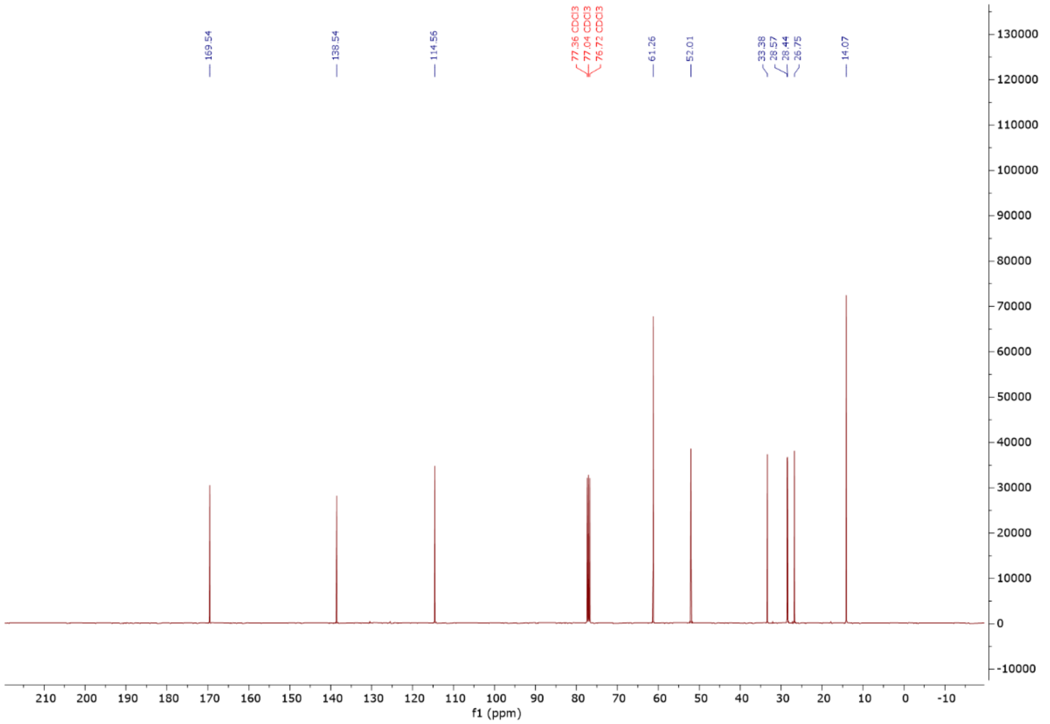

2-(Hex-5-enyl)propane-1,3-diol

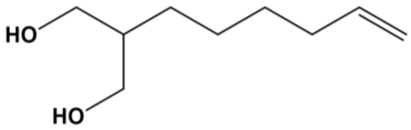

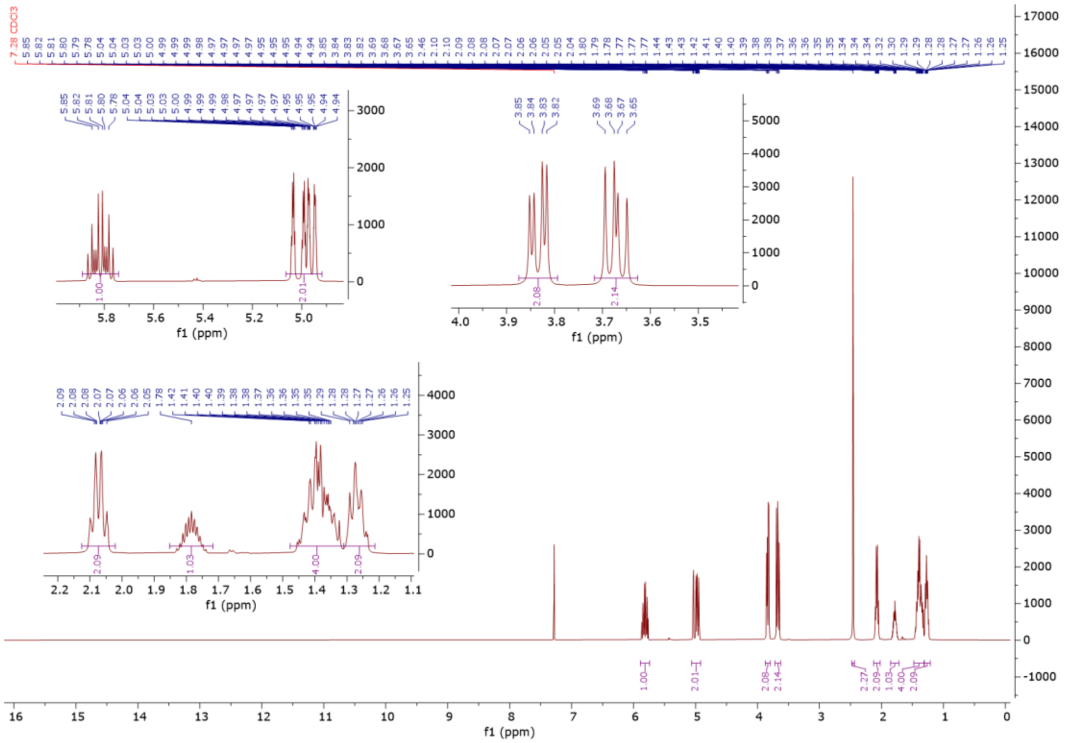

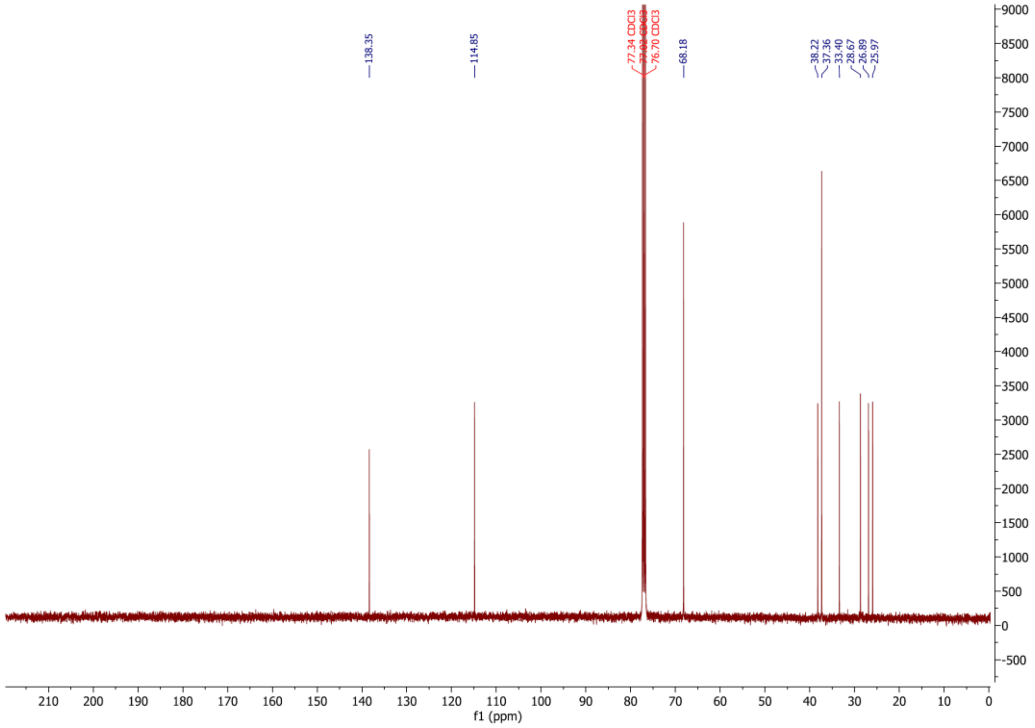

Methanesulfonic Acid 2-Methanesulfonyloxymethyloct-7-enyl Ester

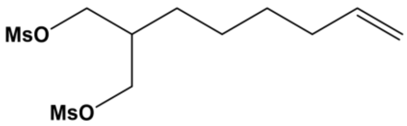

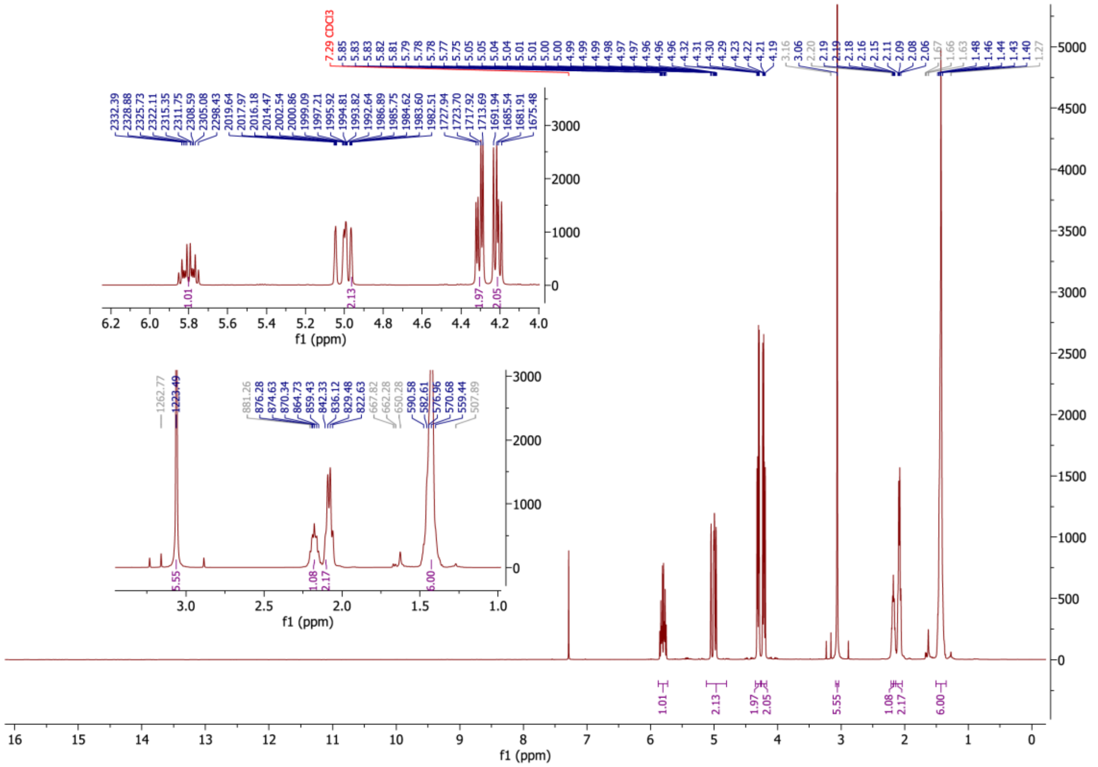

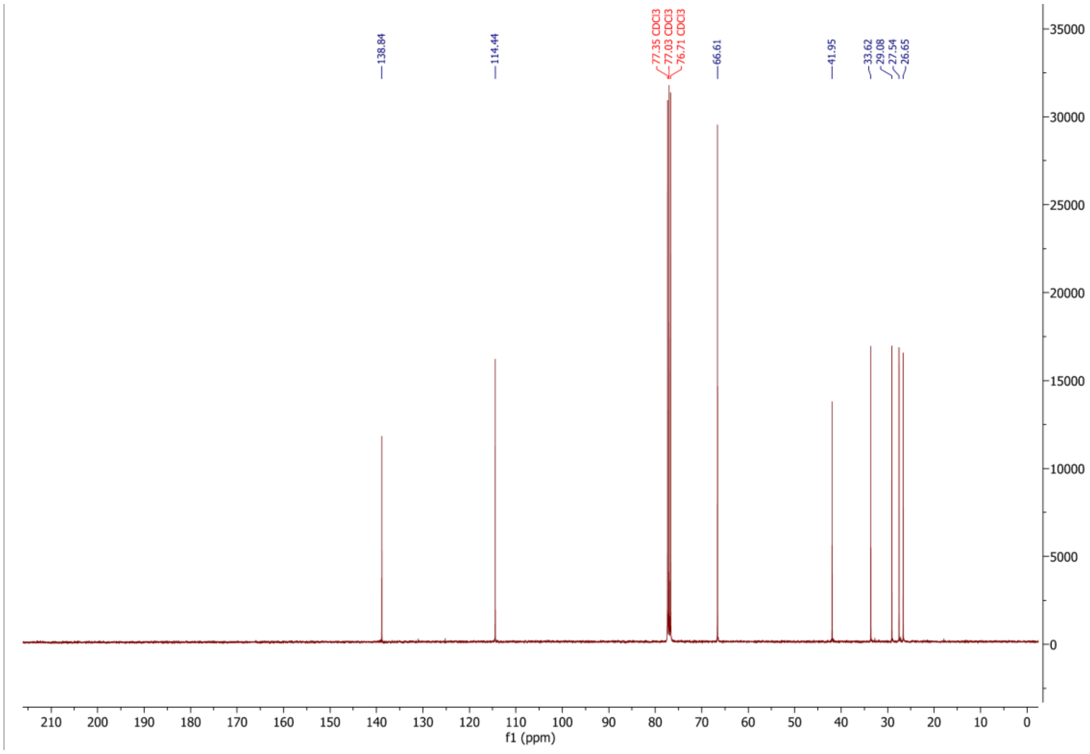

((methylsulfonyl)oxy)-6-(((methylsulfonyl)oxy)methyl)heptanoic acid

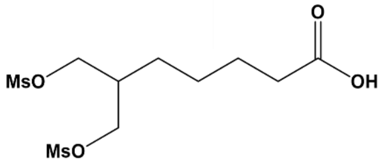

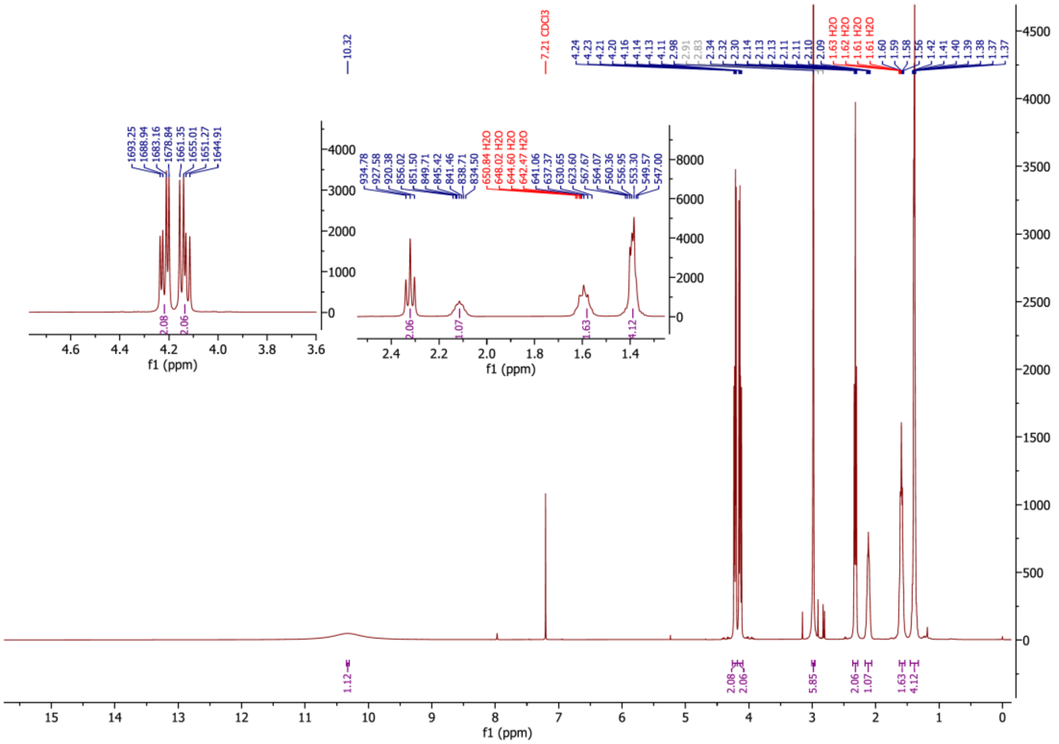

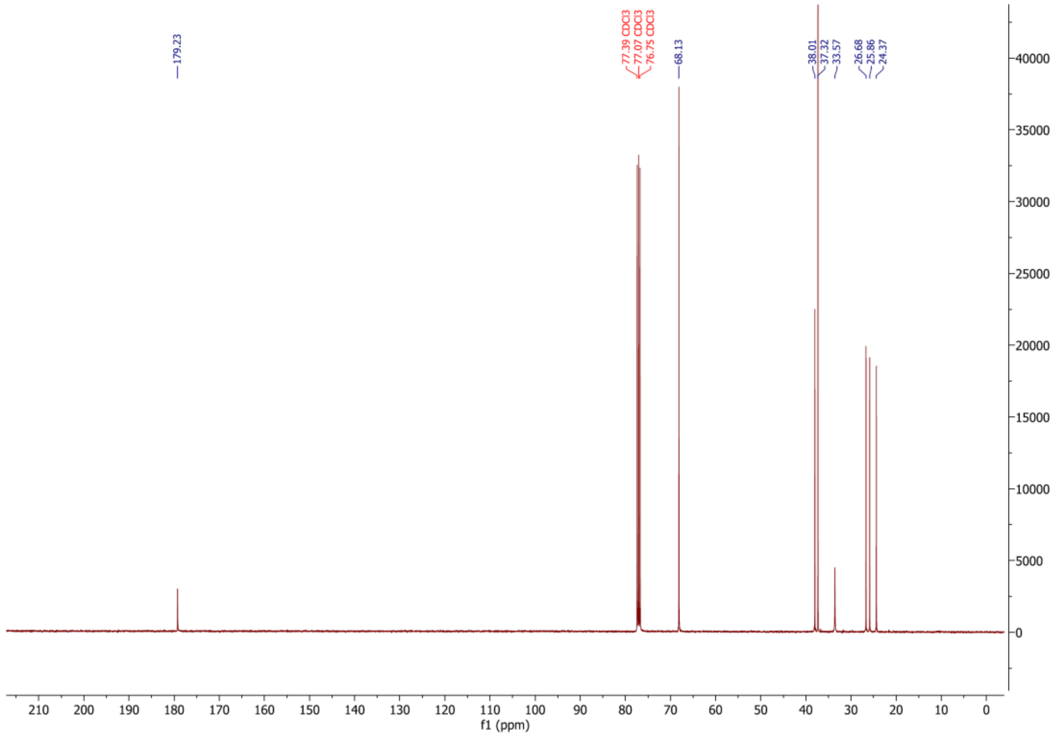

Isolipoic acid

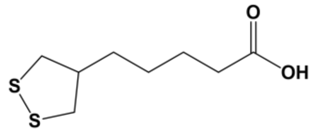

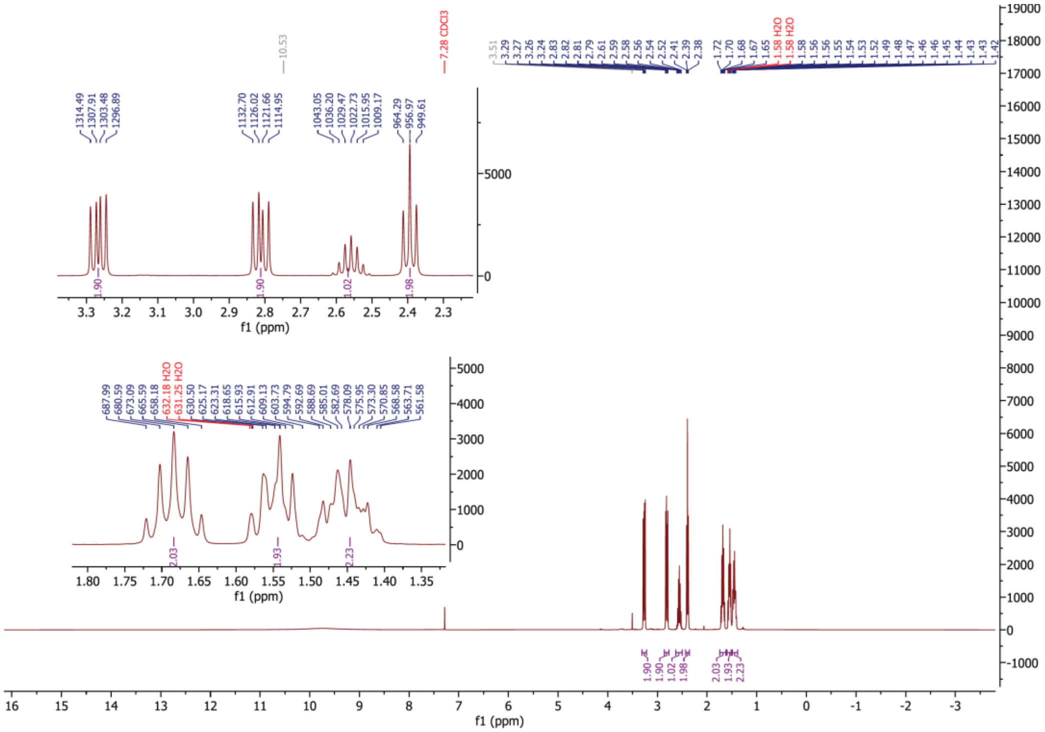

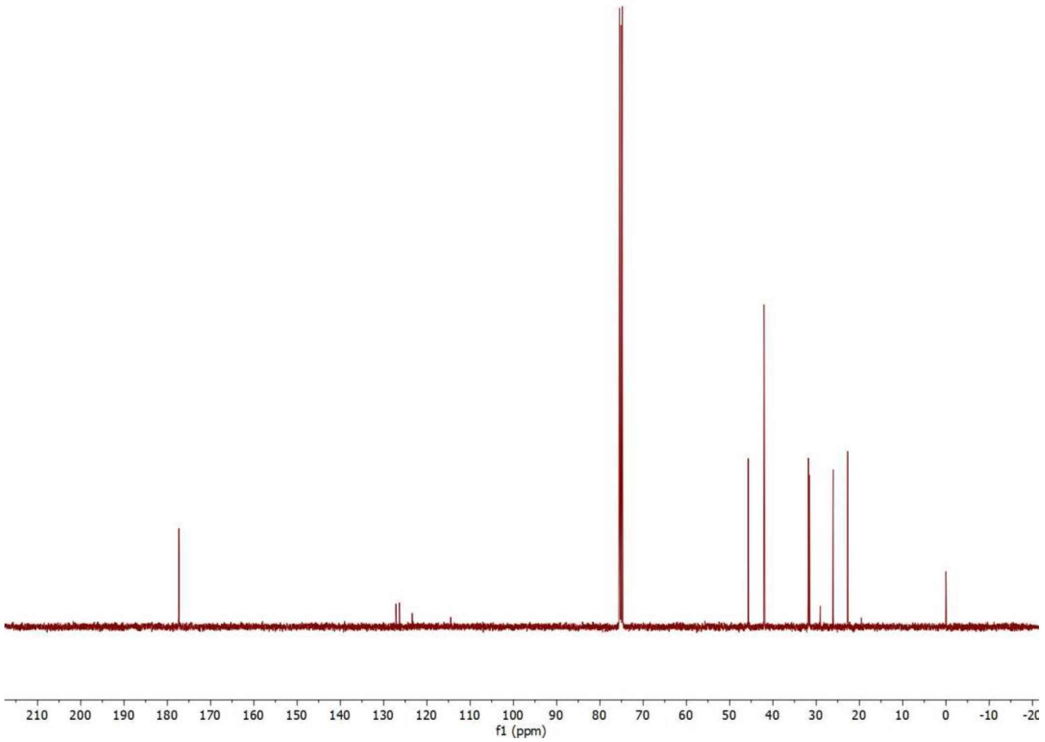

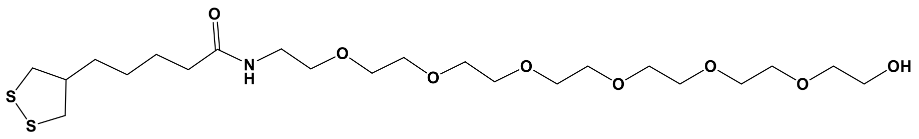

Compound 11

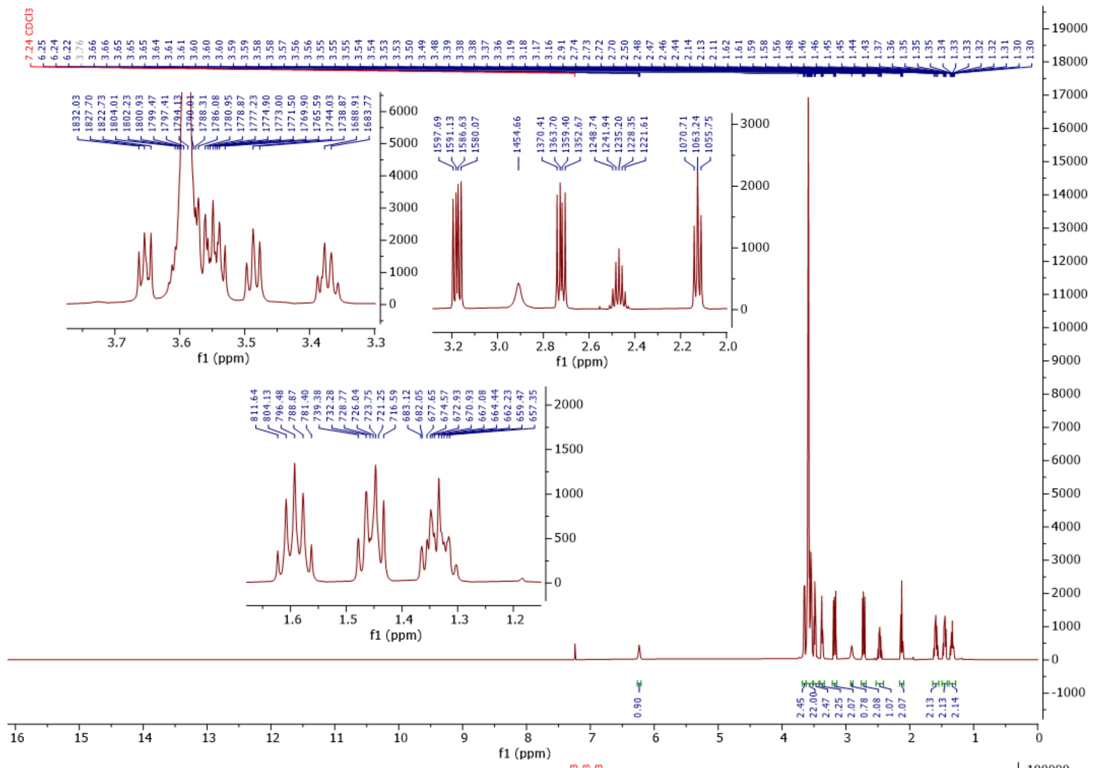

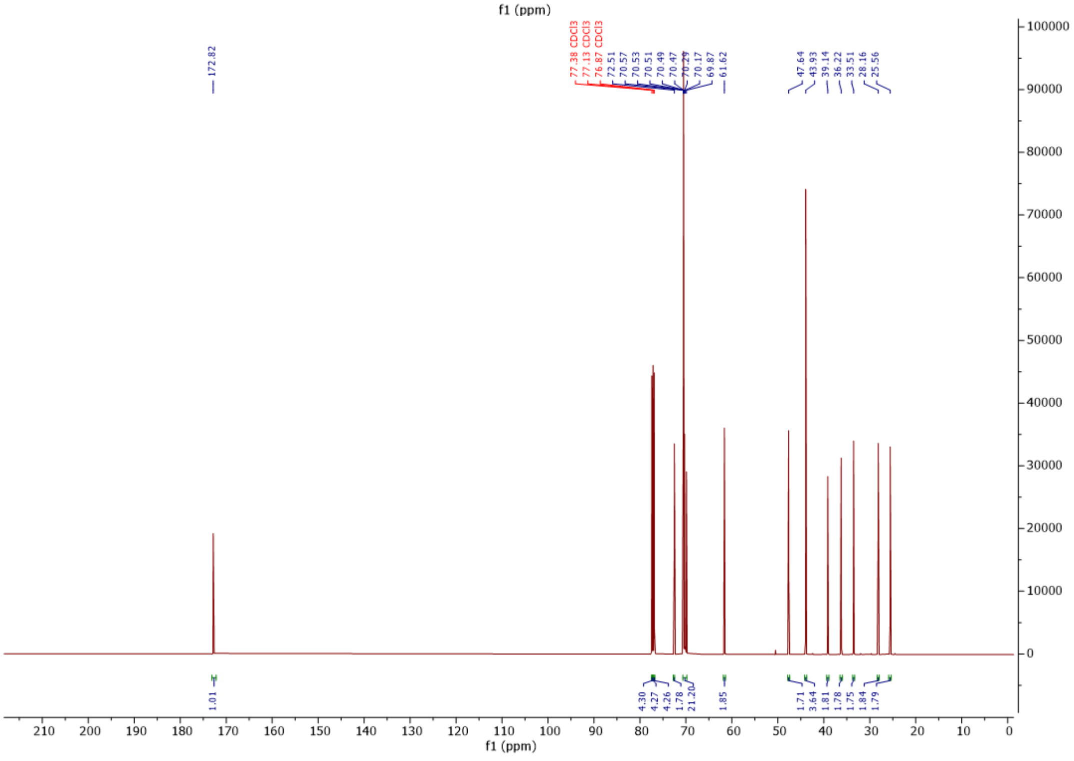

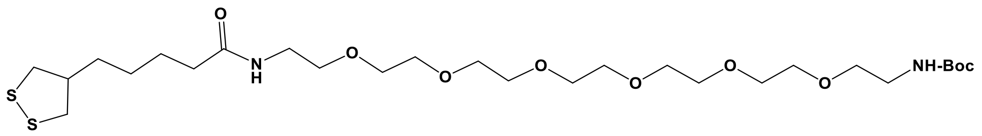

Compound 2

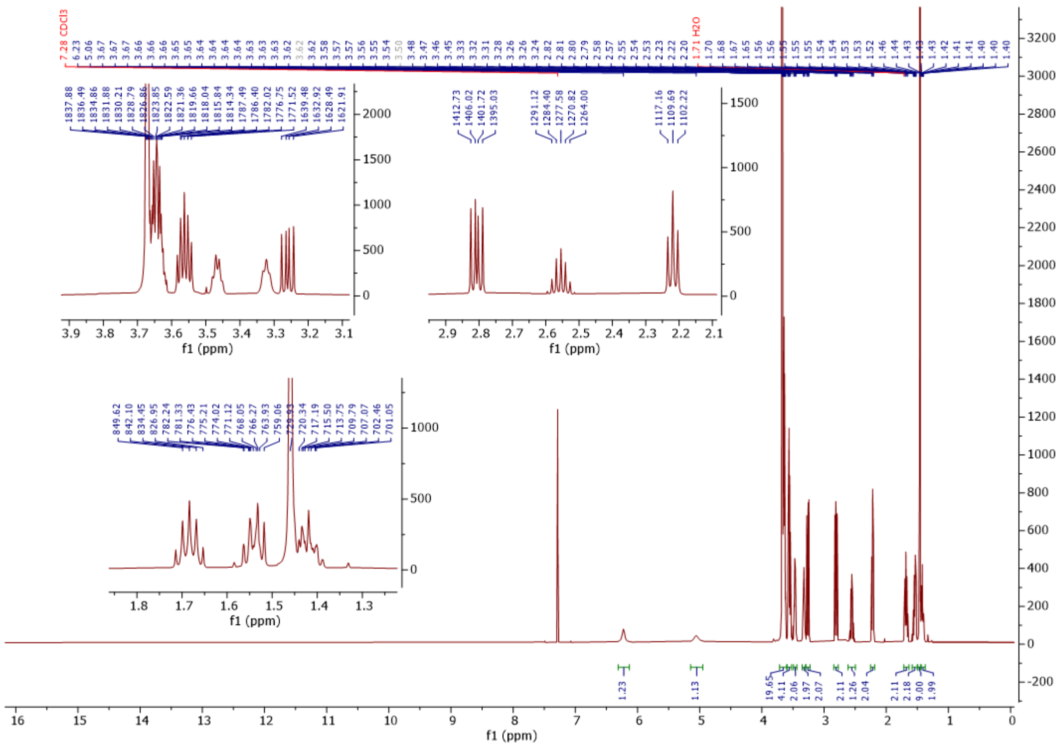

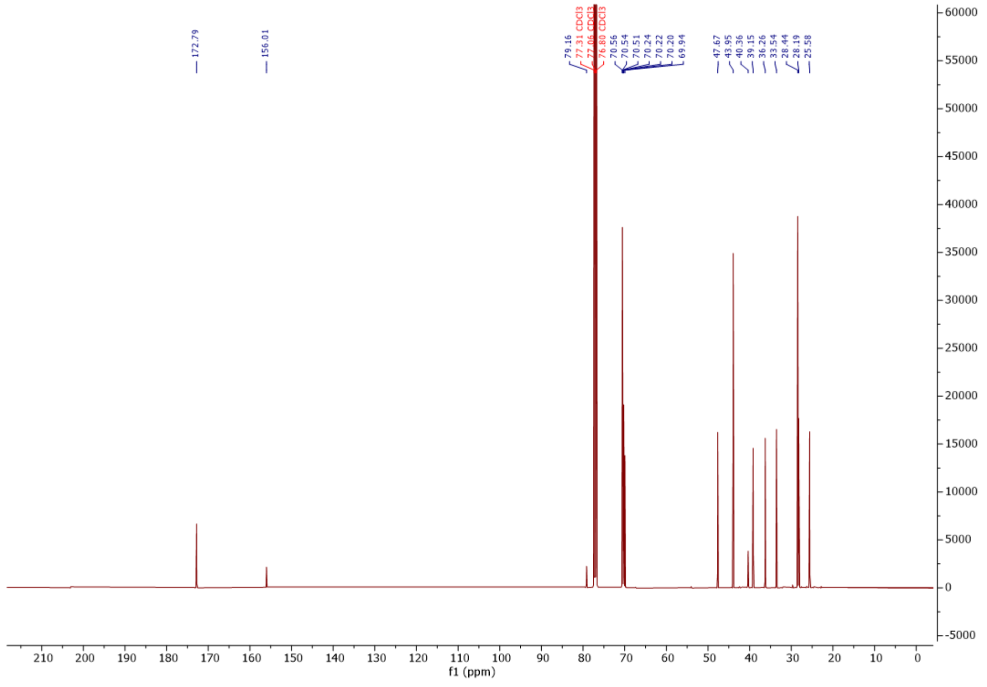

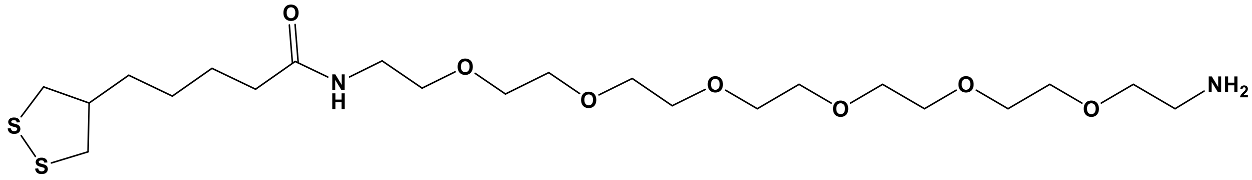

Compound 3

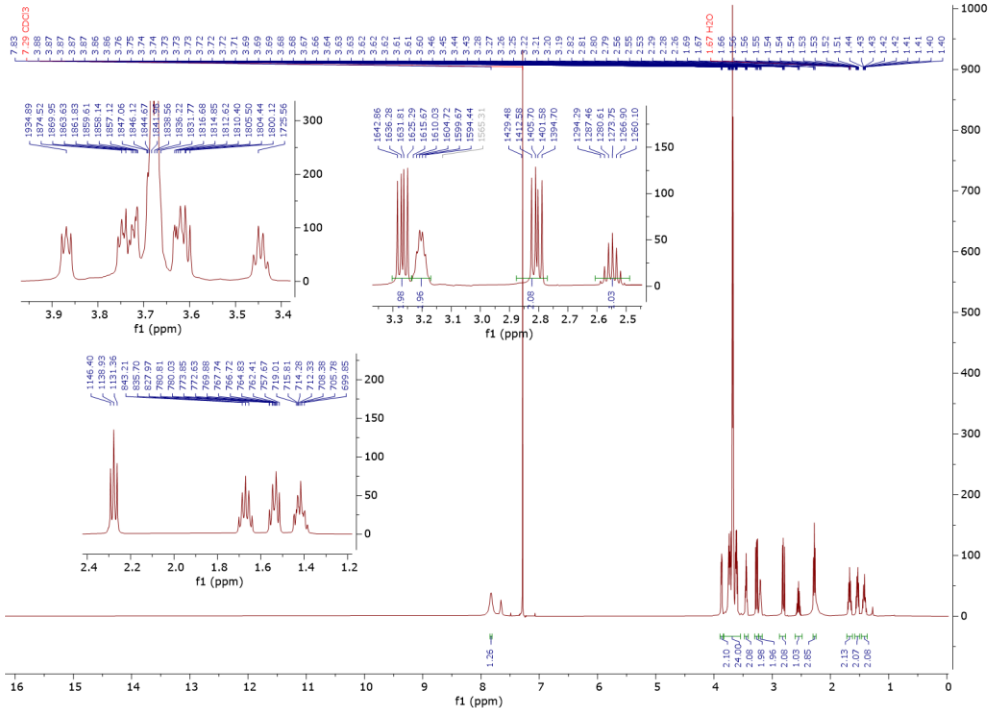

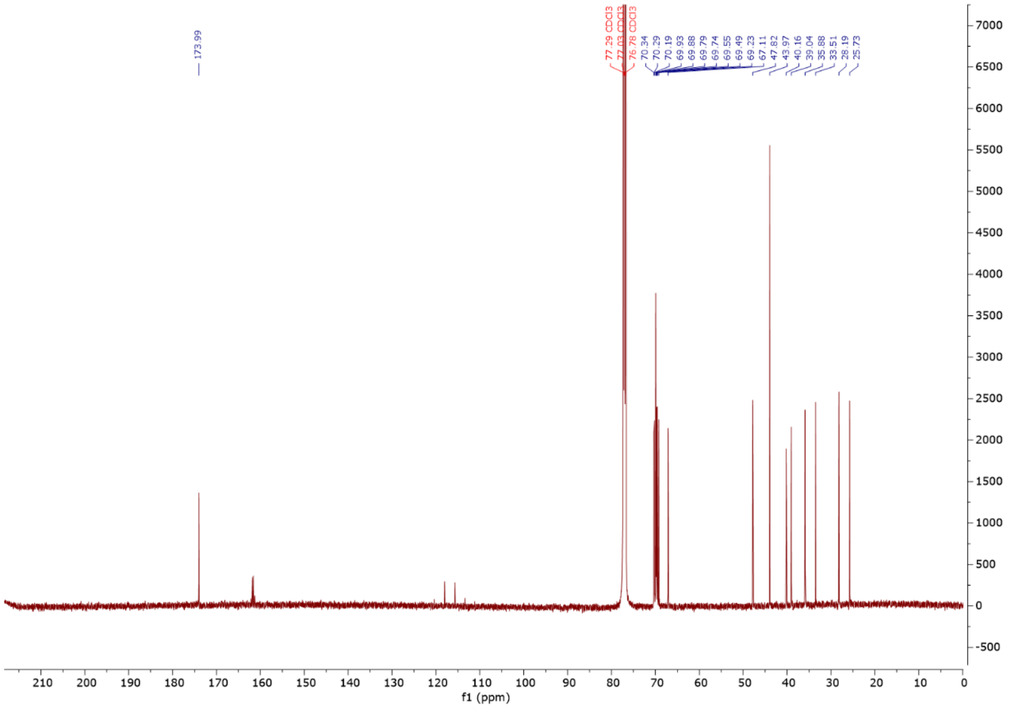

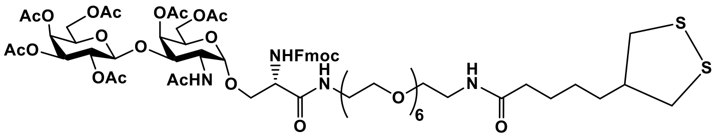

Compound 4

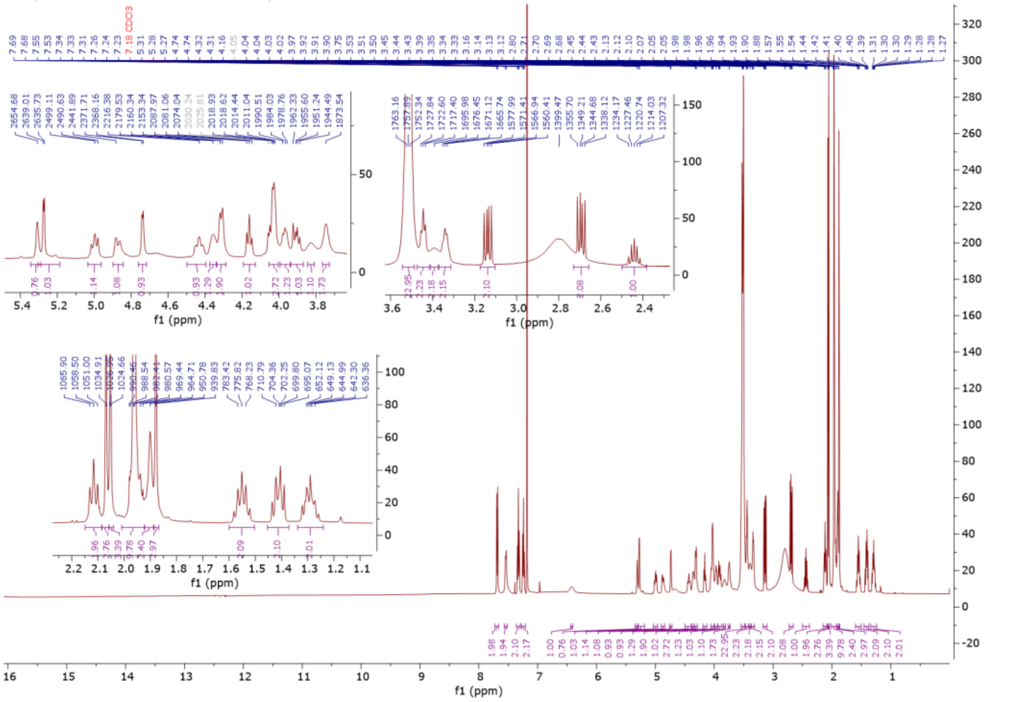

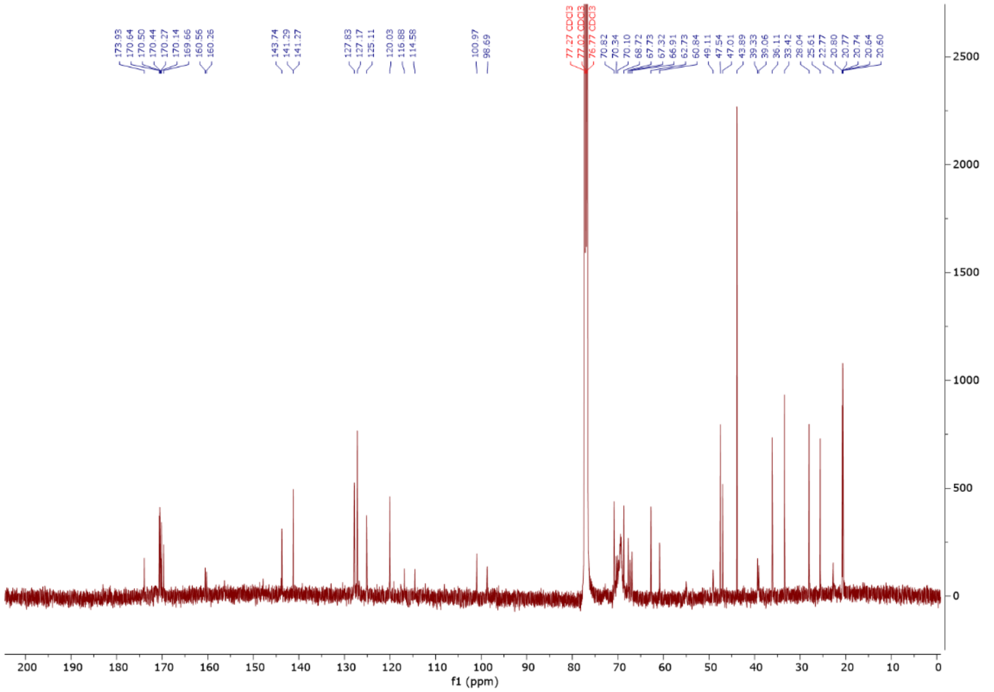

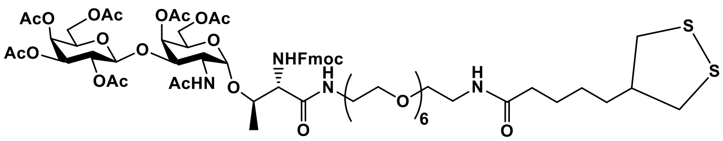

Compound 5

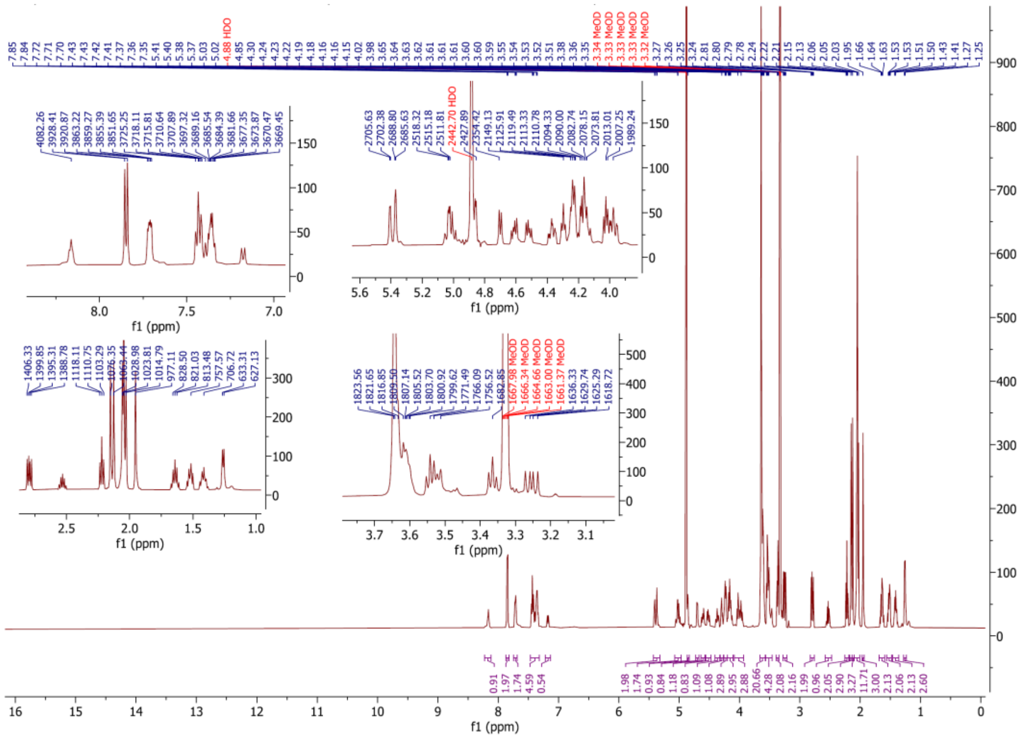

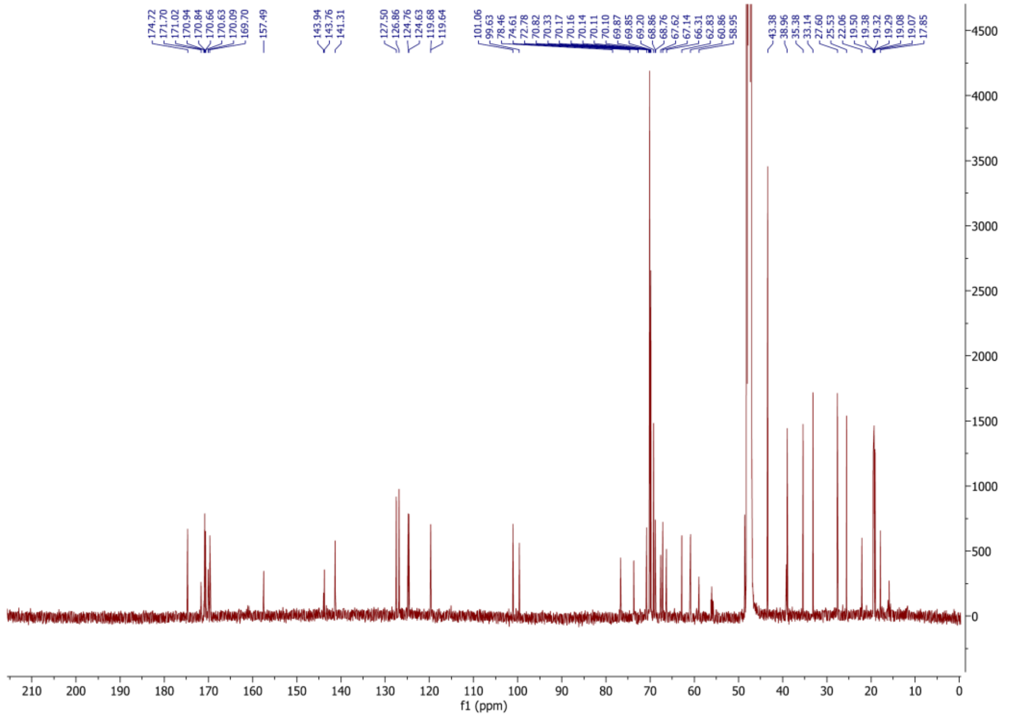

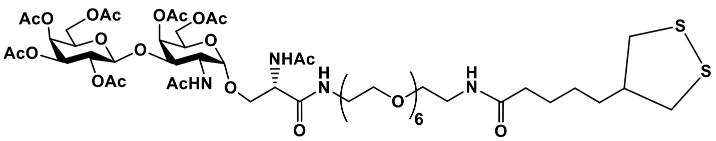

Compound 6

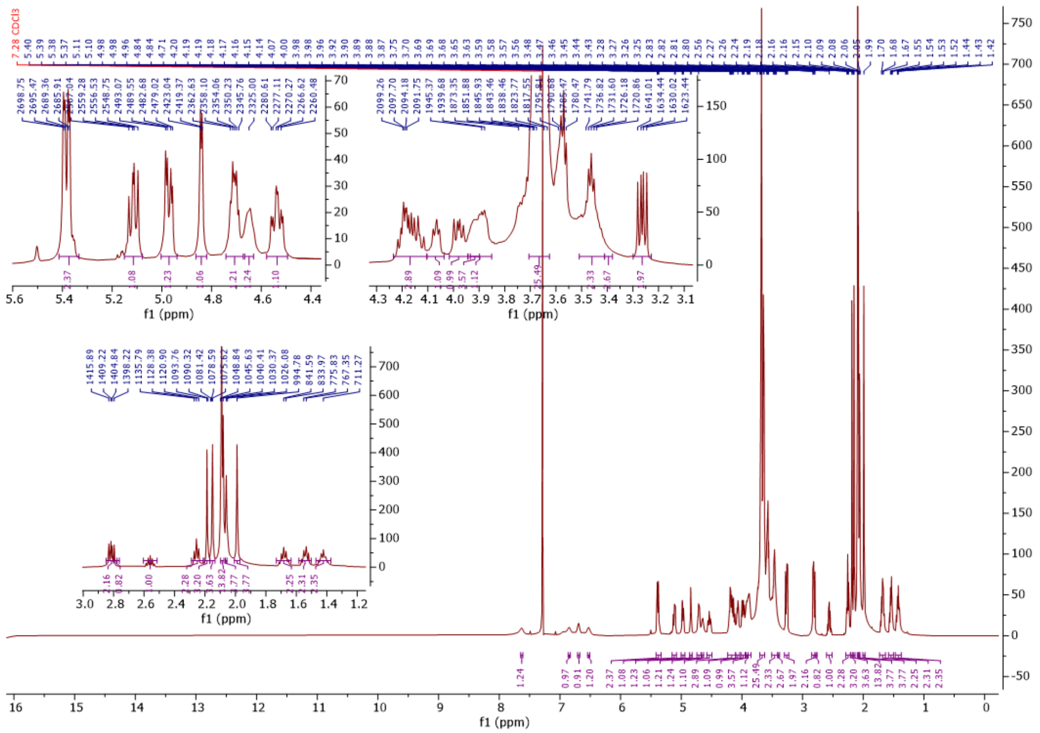

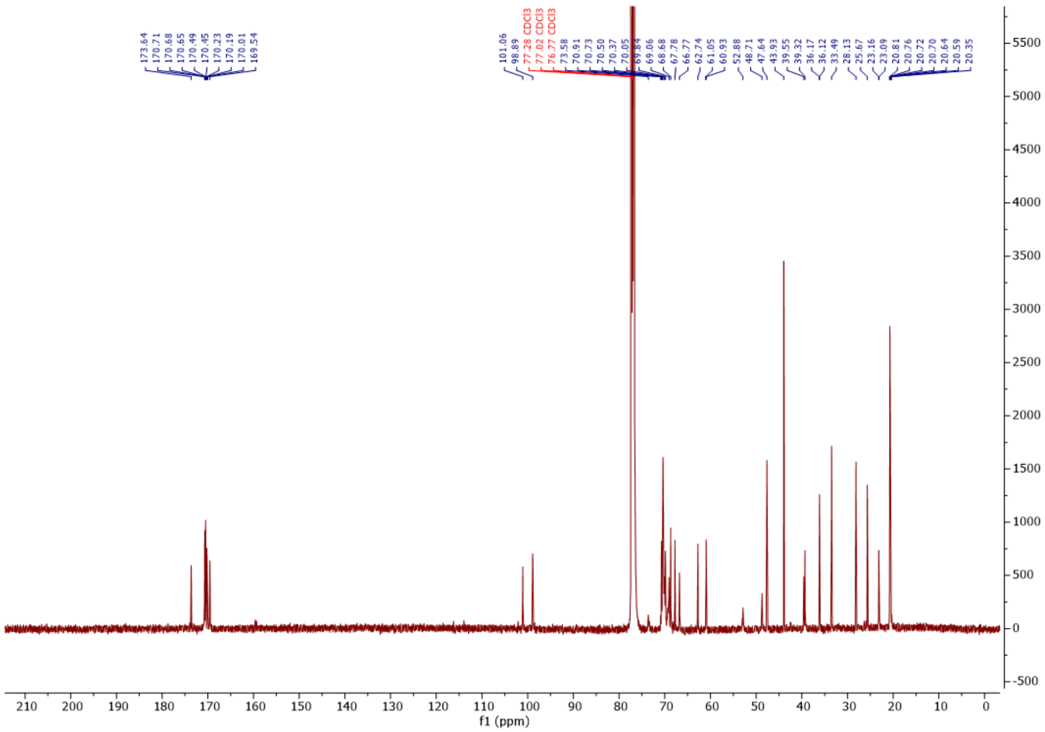

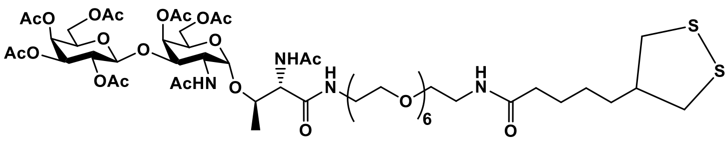

Compound 7

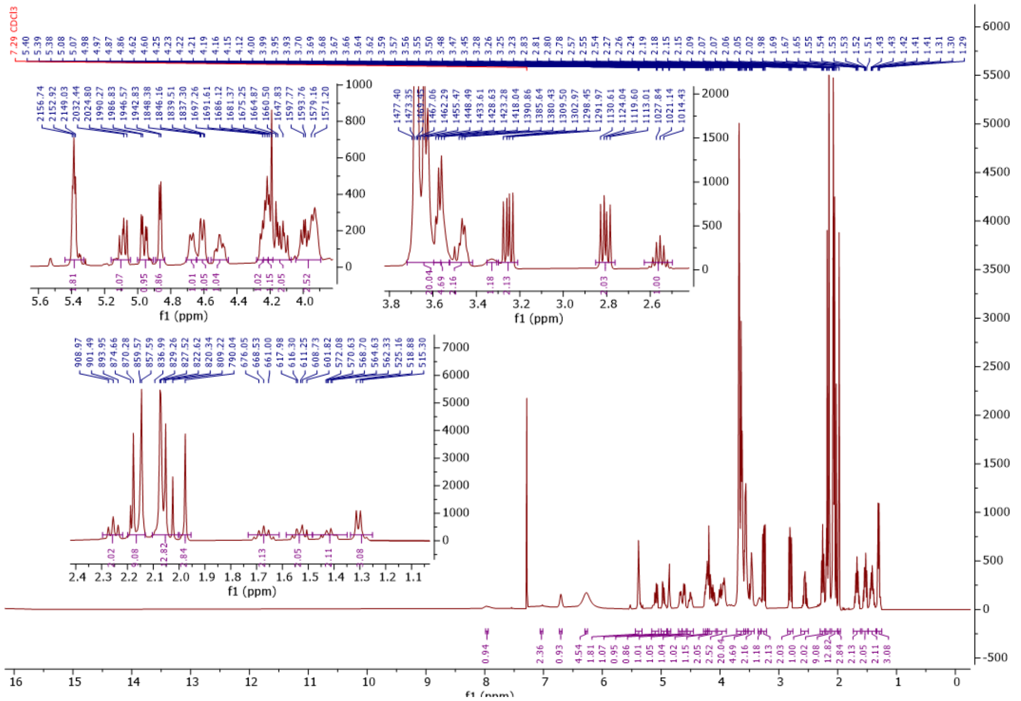

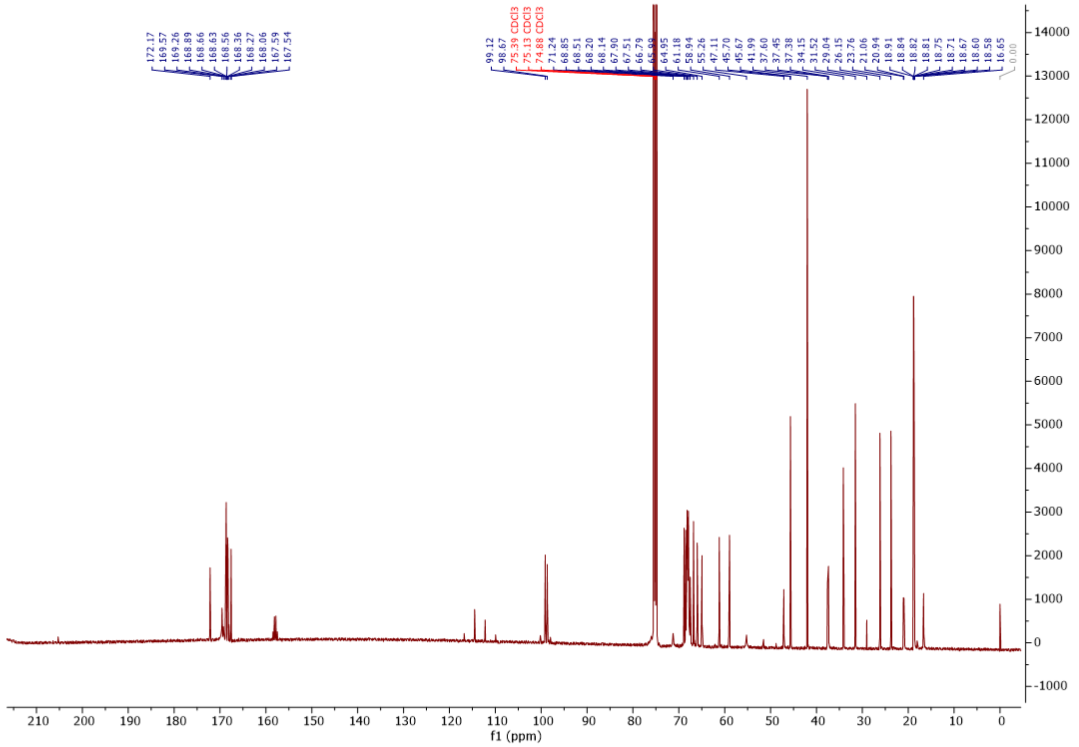

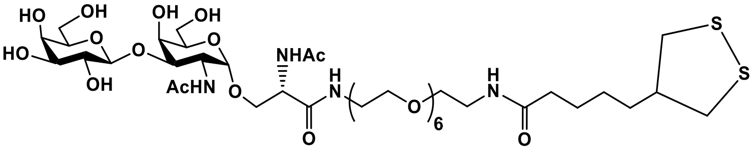
HO
OH
HO
OH
O
OH
OH
O
AcHN
O
NHAc
H
N
O
6
H
N
O
S
S
O

Compound 8

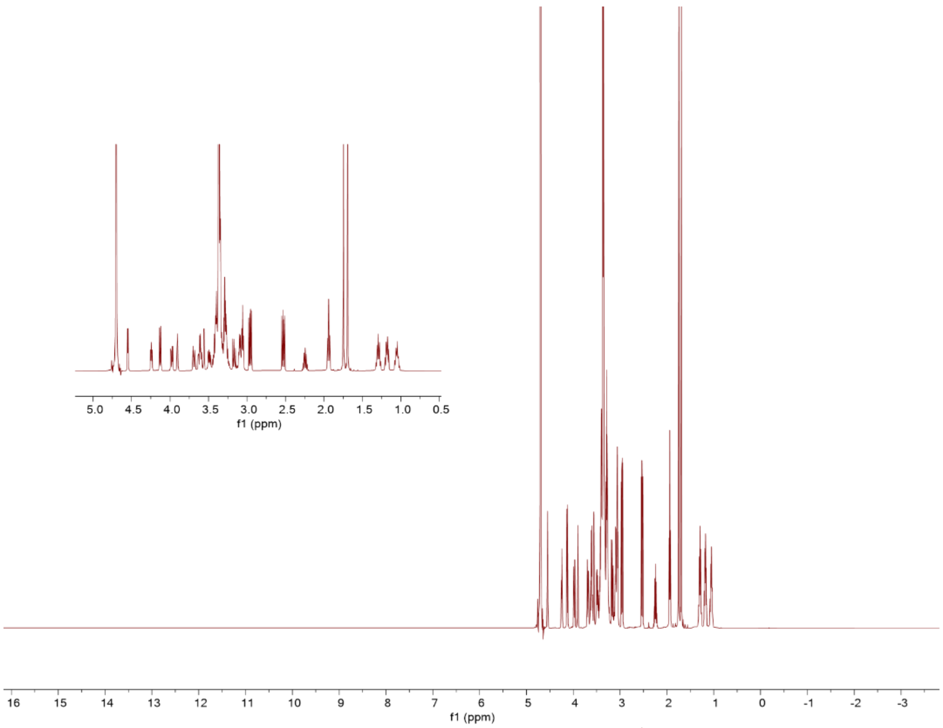
5.0 4.5 4.0 3.5 3.0 2.5 2.0 1.5 1.0 0.5
f1 (ppm)
16 15 14 13 12 11 10 9 8 7 6 5 4 3 2 1 0 -1 -2 -3
f1 (ppm)

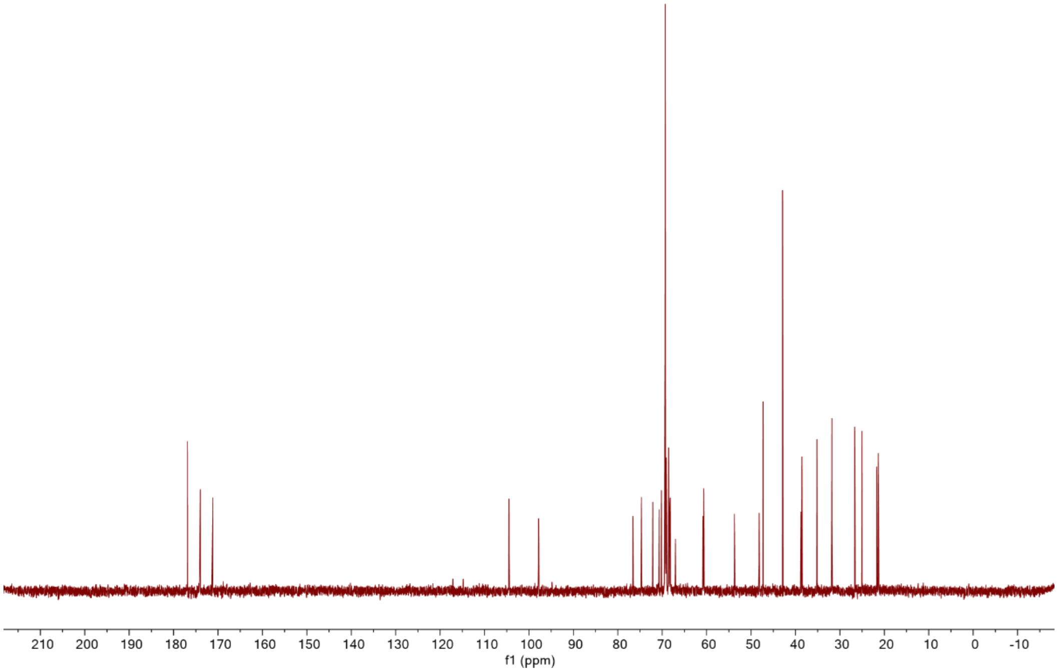
210 200 190 180 170 160 150 140 130 120 110 100 90 80 70 60 50 40 30 20 10 0 -10
f1 (ppm)

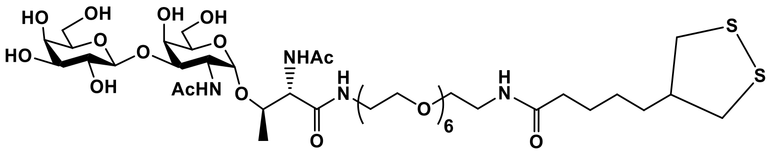

Compound 9

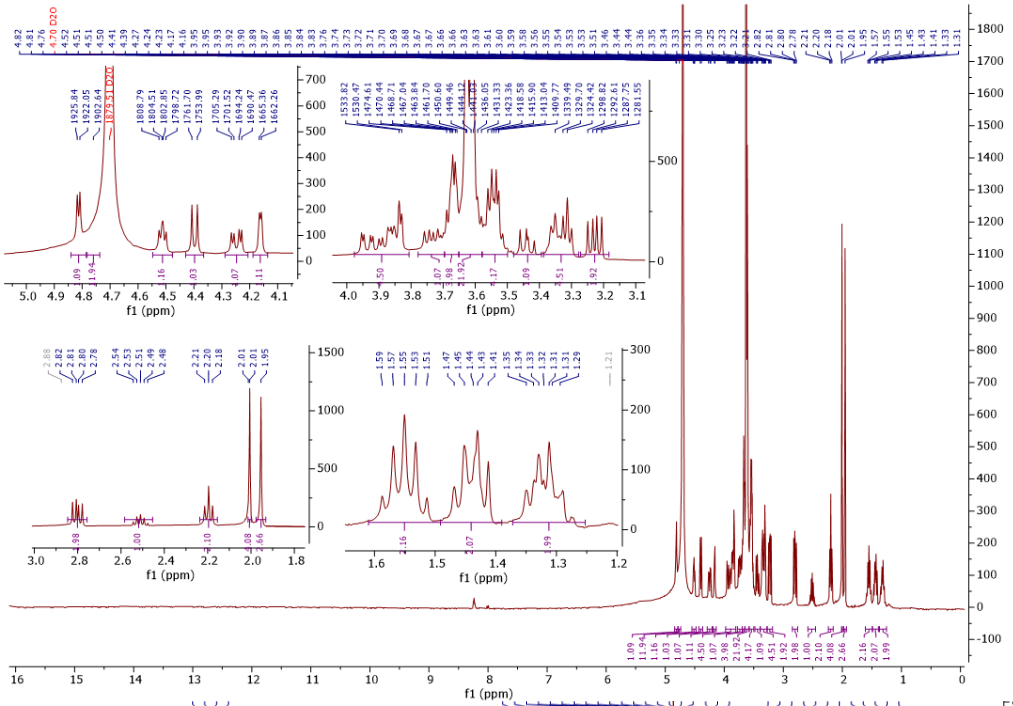

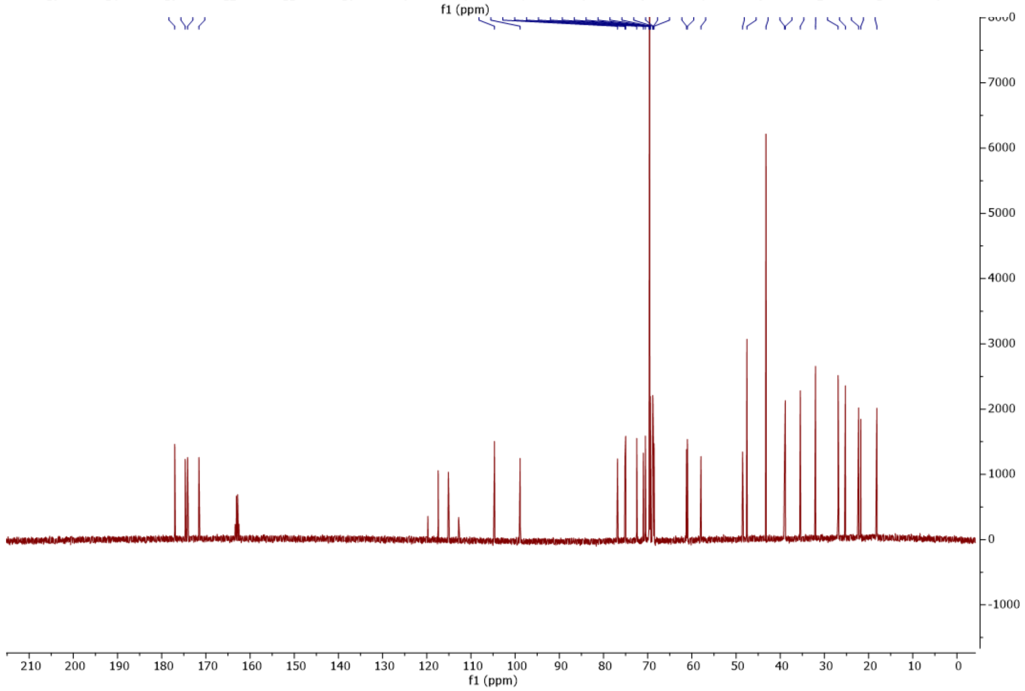

References

1. Boden, S.; Wagner, K.G.; Karg, M.; Hartmann, L. Presenting precision glycomacromolecules on gold nanoparticles for increased lectin binding. *Polymers-Basel* **2017**, *9*.
2. Gupta, A.; Moyano, D.F.; Parnsubsakul, A.; Papadopoulos, A.; Wang, L.S.; Landis, R.F.; Das, R.; Rotello, V.M. Ultrastable and biofunctionalizable gold nanoparticles. *Acs Appl Mater Inter* **2016**, *8*, 14096-14101.
3. Laaf, D.; Bojarova, P.; Pelantova, H.; Kren, V.; Elling, L. Tailored multivalent neo-glycoproteins: Synthesis, evaluation, and application of a library of galectin-3-binding glycan ligands. *Bioconjugate Chem* **2017**, *28*, 2832-2840.
4. Richards, S.J.; Keenan, T.; Vendeville, J.B.; Wheatley, D.E.; Chidwick, H.; Budhadev, D.; Council, C.E.; Webster, C.S.; Ledru, H.; Baker, A.N.*, et al.* Introducing affinity and selectivity into galectin-targeting nanoparticles with fluorinated glycan ligands. *Chem Sci* **2021**, *12*, 905-910.
